# Free Polymers Skew Polyplex Solution Properties and Confound the Interpretation of Structure–Function Relationships

**DOI:** 10.1021/acspolymersau.6c00080

**Published:** 2026-06-15

**Authors:** Jessica L. Lawson, Kaitlin C. Lesco, Ram Prasad Sekar, Grant Wheeler, S. Kim Ratanathanawongs Williams, Ramya Kumar

**Affiliations:** † Materials Science, 3557 Colorado School of Mines, Golden, Colorado 80401, United States; ‡ Bioenergy Science and Technology Directorate, National Laboratory of the Rockies, Golden, Colorado 80401, United States; § Department of Chemistry, Colorado School of Mines, Golden, Colorado 80401, United States; ∥ Chemical and Biological Engineering, Colorado School of Mines, Golden, Colorado 80401, United States; ⊥ Quantitative Biosciences and Engineering, Colorado School of Mines, Golden, Colorado 80401, United States

**Keywords:** field-flow fractionation, polyplexes, AF4-MD, nucleic acid delivery, gradient copolymers, block copolymers, statistical copolymers

## Abstract

Structure–function relationships for polymeric gene carriers are derived from crude, heterogeneous mixtures of free polymers and polymer–pDNA complexes (polyplexes). Recognizing that our current understanding of polyplex solution properties may be erroneous, we separated free polymers from crude polyplexes to gain important, previously inaccessible perspectives into polymer-mediated pDNA delivery. Free polymers, the numerically dominant species in crude polyplexes, impose characterization and quality control challenges similar to empty viral capsids and lipid nanoparticles. Polyplexes are frequently formed at large stoichiometric excesses of cationic groups in polymers relative to phosphates in pDNA resulting in nitrogen-to-phosphorus ratios between 5–100. Most polymers exist as “free polymers” in dynamic equilibrium with pDNA-bound polymers. For the first time, we quantified the contributions of free polymers to polyplex solution properties and biological performance. We separated free polymers from polyplexes by applying asymmetrical flow field-flow fractionation (AF4), which removes free polymers under gentle laminar flow, minimizing shear stress and keeping polyplexes intact. While batch light-scattering analysis of unfractionated crude polyplexes severely underestimates polyplex size and pDNA loading by 2–4×, AF4-aided removal of free polymers sharpened measurements by eliminating background light-scattering contributions. Polyplexes with 78% free polymers aggravated inflammatory responses in macrophages by 2–20× compared to polyplexes devoid of free polymers. Removing free polymers depressed transfection efficiency by 50% while improving cell viability by 30%; reintroducing free polymers abrogated these contrasts. By separating and quantifying free polymers using AF4, we corrected systematic biases in structure–function trends gleaned from crude polyplexes. For instance, microstructure-dependent trends in pDNA loading reported in our earlier work originated from differences in free polymer content rather than the polymer microstructure itself. Our study establishes that free polymers skew measurements of polyplex properties and biological performance and offers design guidance to navigate trade-offs between transfection efficiency, immunogenicity, and toxicity.

## Graphical abstract

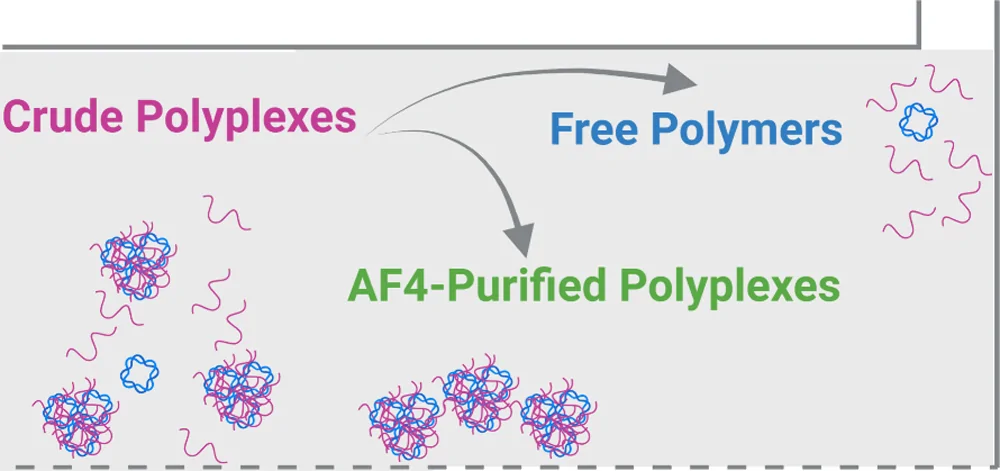

## Introduction

Polymers can overcome intracellular delivery barriers unique to therapeutic nucleic acids such as plasmids (pDNA), mRNA (mRNA), or genome editor proteins.^1^ To evade the crowded intellectual property landscape of lipid nanoparticles (LNPs) and the high manufacturing costs of viral vectors, pharmaceutical companies are turning to polymers. As of 2024, at least 26% of clinical trials in nanocarrier-based therapeutics focus on polymers^2^ and clinical breakthroughs in polymer-mediated gene therapy remain imminent. However, we lack analytical tools that can handle characterization, quality control, and regulatory challenges particular to polyelectrolyte complexes of polymers and nucleic acids (polyplexes). Every measurement—be it polyplex size, pDNA loading, delivery efficiency, or toxicity—is performed not on polyplexes themselves but on heterogeneous mixtures of polyplexes and “free polymers”. To improve colloidal stability and delivery efficiency, researchers add large stoichiometric excesses of polycations to pDNA to achieve nitrogen-to-phosphorus ratios (N/P ratios) in the range of 5–100.^3^ However, most polycations do not bind to pDNA at any instant but instead exist as “free polymers”, which dynamically exchange with pDNA-bound polycations.^4^ Consequently, free polymers easily outnumber polyplex particles in therapeutic formulations, and yet the impact of free polymers on polyplex solution properties and biological performance has never been quantified. Currently, researchers average the solution properties across polyplexes *and* free polymers without estimating their relative abundance. For instance: What is the proportion of free polymers to bound polymers? How does this change with polymer microstructure and composition? Questions like these require bioanalytical tools tailored to the fragility, heterogeneity, and complexity of polyplexes.

Similar to free polymers, empty viral capsids^5–7^ and empty LNPs^8–11^ also pose characterization difficulties. Empty viral capsids, empty LNPs, and free polymers are expected to exacerbate toxicity and immunogenicity,^8,12,13^ especially in high-dosage clinical applications like Duchenne’s muscular dystrophy (DMD). Recently, Sarepta Therapeutics halted their DMD clinical trials of their viral vector formulation, Elevidys, because two patients succumbed to severe inflammation and liver failure due to the high viral titers administered.^14^ Whether free polymers trigger as much inflammation and toxicity as empty LNPs and empty viral capsids,^13,15–18^ and whether free polymers also require careful quantification and removal from therapeutic formulations is an open question with outstanding clinical relevance.

Researchers developed diverse approaches to quantify the prevalence of empty viral capsids or empty LNPs and to remove them.^19,20^ Recently, Mao and co-workers enumerated empty LNPs, estimated mRNA loaded per LNP,^10,21^ and revealed that LNPs are far more heterogeneous than previously believed. Because Mao et al. employed fluorescent labeling of lipids and nucleic acids, there is a need for label-free approaches to capture the heterogeneity in size, mRNA loading, and enumerate empty particles in LNP formulations. Importantly, approaches perfected for viral vectors and LNPs falter when applied to polyplexes, which are far more dynamic than enveloped formulations. It is well-known that polymers and pDNA constantly exchange between individual polyplexes, rendering biophysical analysis challenging.^22–24^ Additionally, current platforms such as dynamic light scattering (DLS) and static light scattering (SLS) ignore background light scattering contributions emanating from free polymers, which can bias estimates of polyplex size and pDNA loading.^25,26^ Although analytical ultracentrifugation can separate empty LNPs from mRNA-loaded LNPs,^11^ high shear stresses will rupture fragile samples like polyplexes.^10,27^ Centrifugation subjects polyplexes to shear stresses several orders of magnitude more intense than in blood circulation.^28,29^

Considering the sample damage introduced by centrifugation and the measurement bias introduced by batch light scattering, we turned to asymmetrical flow field-flow fractionation (AF4) coupled with online detectors (multiangle light scattering or MALS, differential refractive index or dRI, and dynamic light scattering or DLS).^30–32^ AF4 is well-suited to fragile polyplexes because we can separate free polymers from “crude” polyplexes under a laminar flow regime. Compared to centrifugation, AF4 minimizes shear stress by 6 orders of magnitude (Section S4 in the SI) and yields intact purified polyplexes devoid of free polymers for unbiased compositional analysis (Figure 1). We applied AF4 to quantify the contributions of free polymers to polyplex solution properties and intracellular pDNA delivery.^33–36^ Unlike batch MALS analysis of crude polyplexes, online MALS estimates of AF4-purified polyplexes were not skewed by background contributions arising from free polymers. AF4-MALS offers unprecedented insight into structure–function relationships that were earlier missed by conventional measurements of crude polyplexes. Further, AF4-aided removal of free polymers followed by the readdition of modified polymers can enable orthogonal control over pDNA delivery efficiency, toxicity, and inflammation.

**Figure 1:**
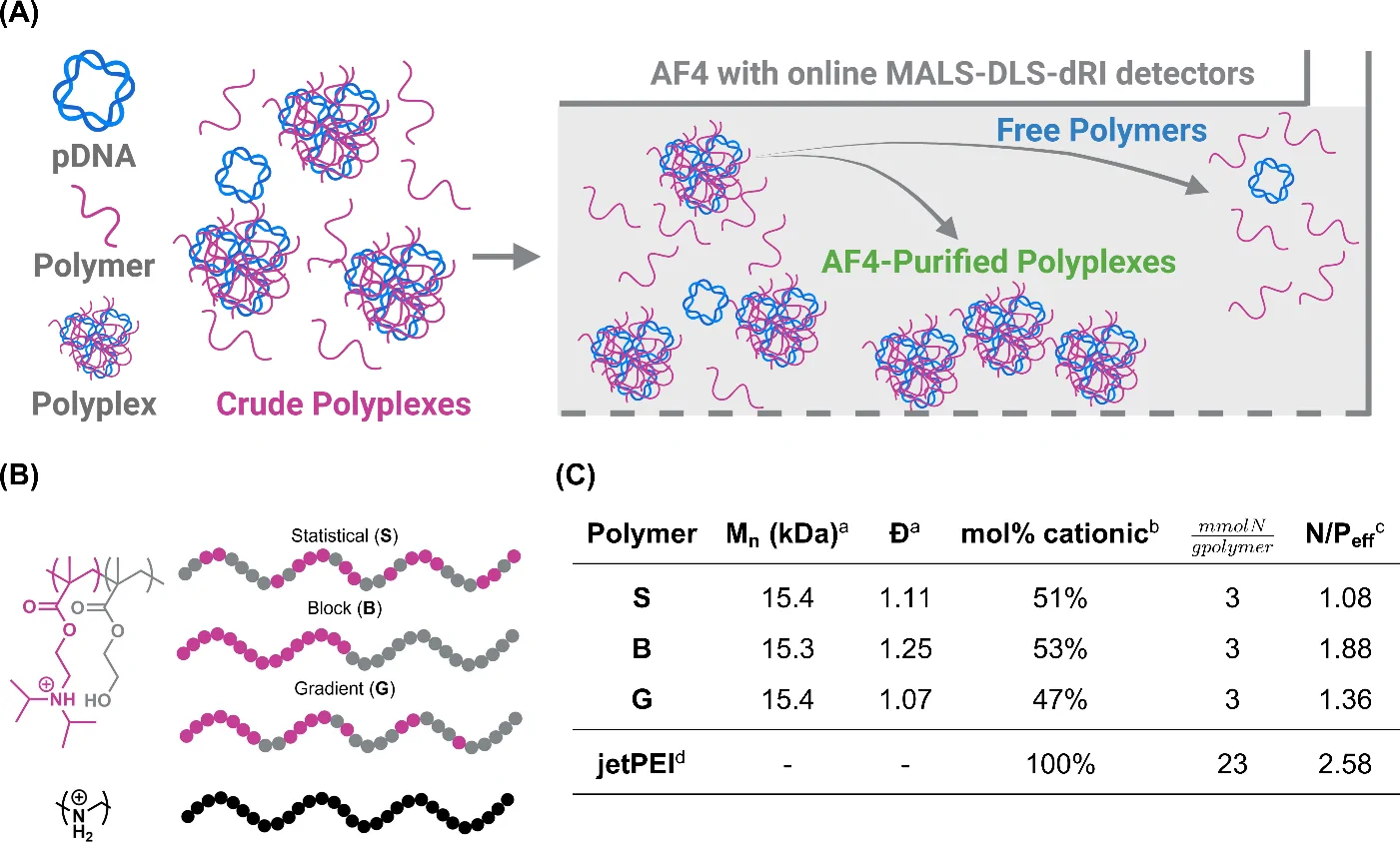
Free polymers dominate polyplexes, confounding the interpretation of solution properties and biological performance. (A) We asked how fractionating free polymers from crude polyplexes via AF4 transformed structure–function relationships in polymer-mediated pDNA delivery. (B) We studied three compositionally equivalent polymers with varying spatial distributions of cations (statistical **S**, block **B**, and gradient **G**), and jetPEI, which has a far higher charge density than the cationic methacrylate polymers. (C) **S**, **B**, and **G** had similar molecular weights (*M*_n_), cationic incorporation, (mol % cationic), and cation charge densities (mmol of nitrogen per gram polymer). ^a^From SEC-MALS, ^b^from ^1^H NMR, ^c^from AF4-MALS, ^d^the exact structure of jetPEI is unknown, linear PEI is drawn as a representation. Created with Biorender.com.

## Results and Discussion

We asked three questions: (1) how do free polymers distort the solution properties of polyplexes that we measure via light scattering, (2) how do free polymers alter the immune response (cytokine secretion) in macrophages in response to polyplexes, and (3) how does the removal and controlled readdition of free polymers affect transfection efficiency and toxicity? To discover how polymer microstructure impacts the partitioning of polycations between free and pDNA-bound states, we selected three composition- and length-matched polymers (Figure S1) with varying microstructure (statistical **S**, block **B**, and gradient **G**).^37^ We also included a commercially available polyethylenimine jetPEI, which has a much higher cationic charge density than **S**, **B**, and **G**, and helped us test whether our approach is generalizable across polymers beyond cationic methacrylates (Figure 1). Thus, we elucidated how microstructural contrasts (**S** vs **B** vs **G**) and chemical differences (jetPEI vs the rest) dictate pDNA loading per polyplex and the ratio of bound to free polymers. Because AF4 exploits differences in diffusivity between polyplexes and free polymers (Figure S2), our approach is agnostic to polymer architecture (e.g., dendrimers, branched polymers, and bottlebrushes) and backbone chemistry (poly­(β-amino ester)­s, polypeptides).

### Unlike Centrifugation, AF4 Removed Free Polymers from Crude Polyplexes while Conserving Polyplex Size and Morphology

We first tried separating free polymers from crude polyplexes via centrifugation, testing three conditions: 14K RCF, 16.5K RCF, and 19.5K RCF. With 14K RCF, we failed to obtain pellets. Both 16.5K RCF and 19.5K RCF failed to yield intact polyplexes. While polyplex radii increased dramatically at 16.5K RCF, indicating aggregation, 19.5K RCF samples lost 30% of their loaded pDNA, indicating rupture (Figure S3). Transmission electron microscopy (TEM) of centrifuged and crude polyplexes confirmed that centrifugation triggered changes in polyplex size and morphology (Figures S4–S6). Unlike centrifugal separation, where shear stresses approach 3.6 × 10^4^ Pa, polyplexes experience extremely mild shear stresses (0.016 Pa) during AF4 (see Section S4 in the SI). Because polyplexes are separated from free polymers under laminar flow regimes, rather than turbulent regimes, shear stresses are reduced by almost 6 orders of magnitude in going from centrifugation to AF4. Unlike chromatography, where the stationary phase entraps and aggregates polyplexes,^38^ AF4 recovers most polyplexes (75–90% mass recovery) while preserving polyplex size, pDNA loading, and morphology (Figures S5–S7).

During AF4, faster-diffusing smaller species such as free polymers counteract the crossflow and diffuse toward the center of the channel. Slower-diffusing larger species such as polyplexes will remain near the semipermeable membrane (Figure S2). Because the channel flow adopts a parabolic velocity profile characteristic of laminar flow regimes, smaller species that occupy regions of faster flow will elute faster than larger species, resulting in shorter retention times into online multiangle light scattering (MALS), dynamic light scattering (DLS), and differential refractive index (dRI) detectors. While the MALS signal is correlated to species size, the dRI signal is sensitive to sample concentration but not size. The dRI and MALS fractograms of **S** revealed two distinct well-separated populations: the first fraction (blue box in Figure 2A) had an intense dRI signal but a weaker MALS signal and the second fraction (green box in Figure 2A) exhibited the opposite trend. We inferred that the first fraction comprises free polymers with trace amounts of unbound pDNA (Figure S8) and the second fraction comprises purified polyplexes, an inference verified by TEM. The first fraction, dominated by water-soluble polymers, appeared blank in TEM. Polyplexes with size distributions and morphology comparable to crude polyplexes were visible in the second fraction (Figure 2A). Importantly, the sizes of crude and AF4-purified polyplexes were indistinguishable: *R*_*TEM*_ of 42 ± 9 nm and 39 ± 9 nm, respectively (Figures S5–S6).

**Figure 2:**
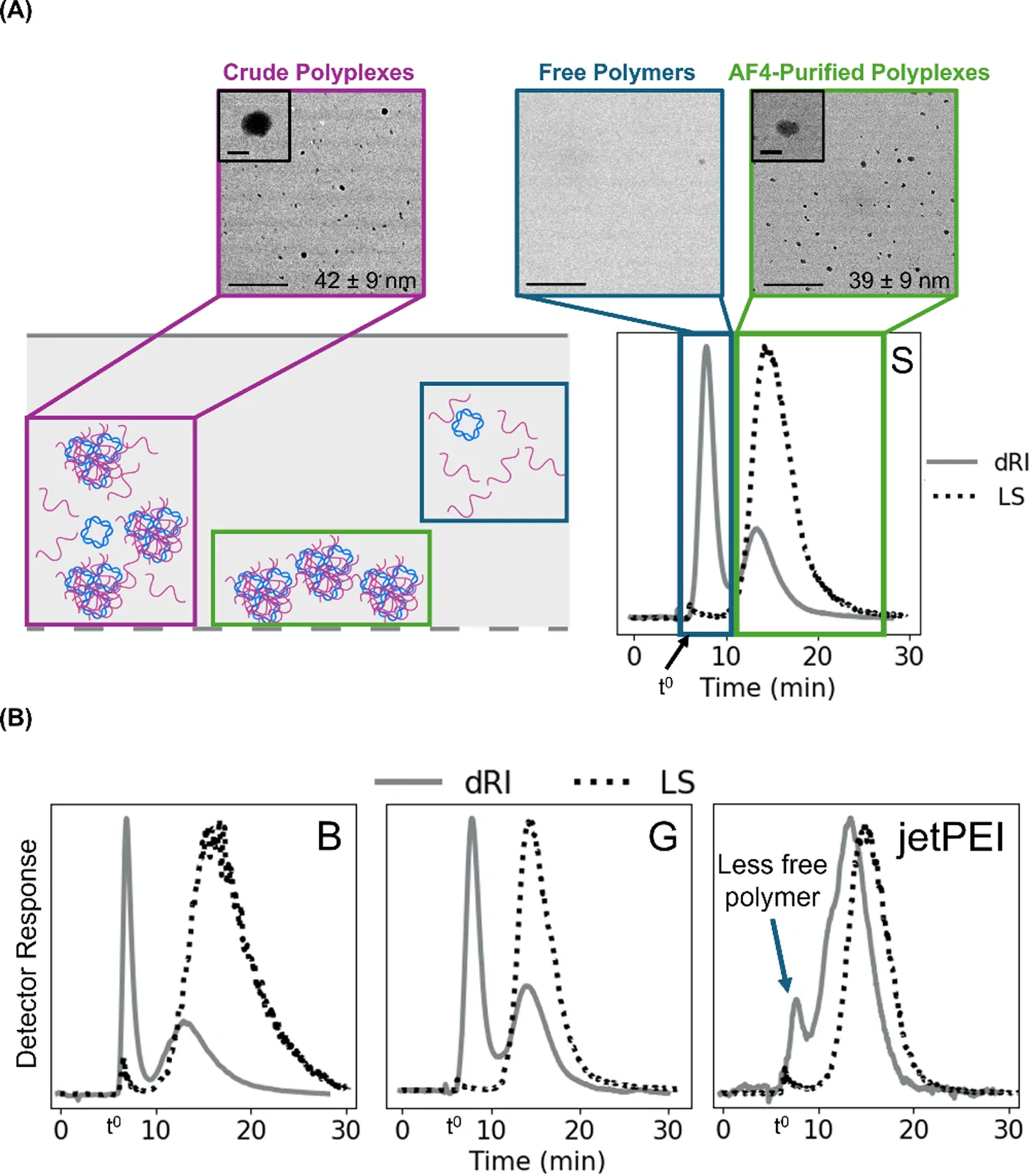
AF4 separated free polymers from crude polyplexes while conserving polyplex structural integrity. (A) TEM imaging confirmed that AF4-purified **S** polyplexes (green box) retained the morphology and size distribution of crude **S** polyplexes (pink box) after removing free **S** polymers (blue box). (B) AF4 also separated free polymers from **B**, **G**, and jetPEI polyplexes, obtaining two distinct well-separated peaks corresponding to free polymers and AF4-purified polyplexes. Interestingly, while the proportion of free to bound polymers was similar across **S**, **B**, and **G** polyplexes, free polymers are far less abundant in jetPEI polyplexes due to its higher effective N/P ratio. Scale bars are 1 μm and 100 nm for TEM images and insets. Scheme created with Biorender.com.

Similar to **S**, AF4 effectively removed free polymers from crude polyplexes for **B**, **G**, and jetPEI (Figure 2B). Interestingly, the jetPEI free polymers’ fraction displayed a much less intense dRI signal compared to **S**, **B**, and **G**, indicating that jetPEI has the lowest proportion of free-to-bound polymers. Free polymers are less dominant for jetPEI compared to **S**, **B**, and **G** due to the higher effective N/P ratio of jetPEI (Figure 1c). Although all polyplexes were formed at an N/P ratio of 5, the effective N/P ratio, which quantifies the actual amount of polymer bound to pDNA in the polyplexes, is far lower. From our previous work with isothermal titration calorimetry (ITC), we estimated effective N/P ratios around 1 for **S**, **B**, and **G**.^37^ Other reports have estimated an effective N/P ratio of ≈ 2.5–3 for jetPEI.^39^ These reported effective N/P ratios are close to values obtained from a rough AF4 mass balance analysis (Figure S8). The effective N/P ratios from ITC are slightly lower than those from AF4 mass balance, especially for **B** (1.9 vs 1). By reducing shear stress by 6 orders of magnitude lower than centrifugation, AF4 gently separated free polymers, yielding structurally intact polyplexes with the same size distribution as crude polyplexes. AF4-MALS analysis of free polymer and purified polyplex fractions revealed that free polymers are somewhat less abundant in polyplexes formed from polycations with high charge densities (such as jetPEI). Having elucidated how polymers’ chemical functionality impacts the partitioning of polycations between free and polyplex-bound states, we next asked whether contrasts in polymer microstructure would yield differentially pDNA-loaded polyplexes.

### Free Polymers Depress Estimates of pDNA Loading Per Polyplex

Several research groups, including ours, routinely apply batch MALS to determine the weight-averaged molecular weight *M*_*w*_ and thus calculate pDNA loading per polyplex.^37,39–42^ However, batch MALS of crude polyplexes yields erroneously low estimates of *M*_*w*_ and pDNA loading (Figure 3) due to background scattering contributions from free polymers. By averaging the *M*_*w*_ values over low molecular weight species such as free polymers and polyplexes, batch MALS systematically underestimates *M*_*w*_ and pDNA loading (Figures 3A and 3B). We applied AF4-MALS to gently remove free polymers and obtain unbiased estimates of polyplex solution properties. In Figure 3C, we compared the *M*_*w*_, *R*_*g*_, and pDNA loading per polyplex and computed the shape factor *R*_*g*_/*R*_*h*_, which sheds light on polyplex sphericity.^34,40^ Shape factor values close to 2 indicate rod-like polyplexes while values closer to 0.7 and 1 indicate spherical polyplexes.

**Figure 3:**
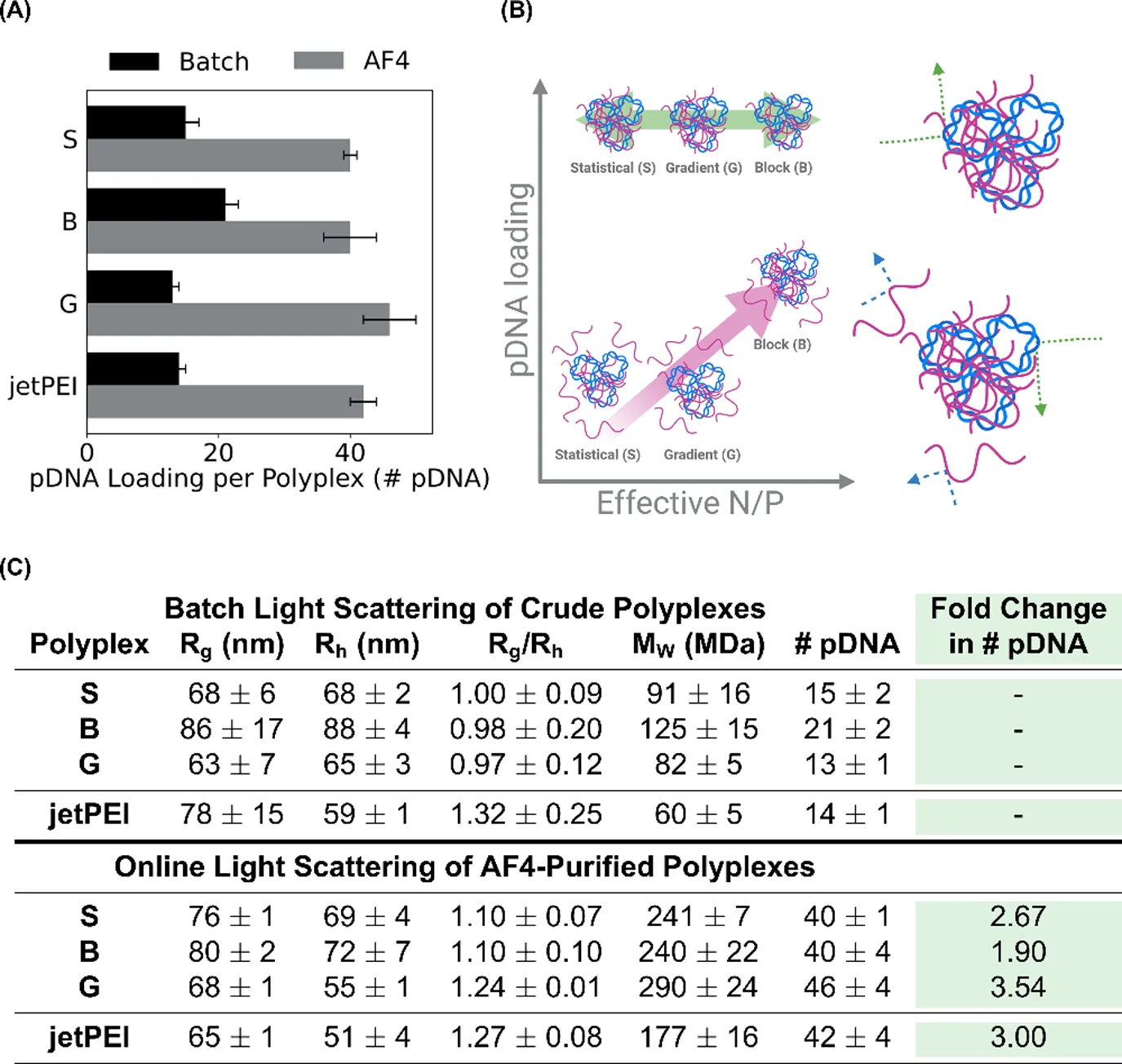
AF4 separation of free polymers corrects our understanding of relationships between polymer microstructure and polyplex solution properties. (A) Batch MALS of crude polyplexes underestimates molecular weight, skewing relationships between polymer microstructure and pDNA loading per polyplex. In contrast, AF4 removes free polymers from polyplexes prior to online MALS, estimating polyplex size and pDNA loading without the confounding background contributions of free polymers. (B) Batch MALS suggested that crude polyplexes formed from **B** loaded more pDNA per polyplex than **S** and **G** (pink arrow). However, these microstructure-dependent trends vanished after AF4 removed free polymers (green arrow). (C) We obtained larger estimates of *M*_*w*_ and pDNA loading for AF4-purified polyplexes than crude polyplexes but *R*_*g*_, *R*_*h*_, and the resulting shape factors (*R*_*g*_/*R*_*h*_) were comparable. Data presented as mean ± standard deviation for *n* = 3. Created with Biorender.com.

We did not note any significant differences between *R*_*g*_, *R*_*h*_, and shape factor between batch MALS of crude polyplexes and online MALS of AF4-purified polyplexes. Because free polymers are relatively small (<20 kDa) and diffuse rapidly, we expected they would depress estimates of *R*_*h*_ in crude polyplexes. However, the hydrodynamic radii for these polymers is ≈5 nm which is below the detection limit for our DLS. Additionally, the *R*_*h*_ for unbound pDNA is comparable to polyplexes (≈70 nm) so neither free polymers nor unbound pDNA significantly altered our estimates of *R*_*h*_ in batch. Further, because free polymers are isotropic scatterers, *R*_*g*_ values remain unaffected by AF4 removal of free polymers from crude polyplexes. Consequently, we observed negligible changes in shape factor (*R*_*g*_/*R*_*h*_) after AF4. Both batch MALS and AF4-MALS estimated shape factors around ≈1, indicative of a loosely packed sphere (Figure 3C). Shape factors from AF4 are consistent with the spherical morphology we repeatedly observe during direct TEM imaging of crude polyplexes (Figure 2B).

Importantly, the *M*_*w*_ values of AF4-purified polyplexes were 2–4× that of crude polyplexes. Consequently, we obtained higher pDNA loading per polyplex for AF4-purified polyplexes compared to crude polyplexes: 40 versus 15 for **S**, 40 versus 21 for **B**, 46 versus 13 for **G**, and 42 versus 14 for jetPEI (Figure 3A). AF4 led us to revisit our previous estimates^37^ of how polymer microstructure affected polyplex solution properties (Figure 3B). Batch MALS (Figure S9) had indicated that **B** polyplexes loaded more pDNA (21) compared to **S** and **G** (15 and 13, respectively).^37^ At that time, this finding was difficult to reconcile physically. After all, **B** polyplexes should not be able to load so much more pDNA than **S** and **G** because all three have identical cationic charge densities, compositions and molecular lengths. However, AF4-aided removal of free polymers yielded comparable pDNA loading for all three microstructures (40–46 pDNA per polyplex), invalidating our earlier conclusion about the superior pDNA loading capacity of **B**. Therefore, AF4 unveils important structure–function trends that were earlier being obscured by the presence of free polymers. Structure–function trends gleaned from solution properties of crude polyplexes may not accurately capture underlying microstructure- or composition-driven differences.

Additionally, we uncovered solution properties unique to jetPEI polyplexes which could explain its capacity to transfect cells efficiently without triggering excessive toxicity. Compared to **S**, **B**, and **G**, the mass ratio of pDNA to polycation is far higher in jetPEI polyplexes. jetPEI has the highest density of cationic groups and the highest effective N/P ratio. Despite loading comparable amounts of pDNA per polyplex as **S**, **B**, and **G**, jetPEI polyplexes, remarkably, minimize the mass ratio of toxic polycations to pDNA payloads. Thus, jetPEI’s high charge density allows this polymer to package pDNA efficiently while using the least amount of polycation possible. We suspect that these unique solution properties allow jetPEI to achieve exceptional pDNA delivery efficiency while avoiding immunogenicity and cell death. Next, we asked how free polymer content in polyplexes impacted inflammatory responses by measuring cytokine secretion in macrophages.

### Free Polymers Elevate the Secretion of Inflammatory Cytokines by Macrophages

We measured tumor necrosis factor α (TNF-α) and chemokine (C–C motif) ligand 2 or CCL-2 secretion (Figure 4) for **S** polyplexes with varying free polymer content (0% to 86%), defined as the ratio of free to bound polymers. In clinical trials, viral and nonviral gene delivery platforms elevated cytokine levels to such an extent that anti-inflammatory drugs had to be administered to contain adverse reactions.^43^ Recognizing the importance of predicting and controlling polyplex immunogenicity, we performed cytometric bead assays to quantify TNF-α, which is secreted by inflammatory macrophage subpopulations, and CCL-2, also an inflammation marker. We compared cytokine secretion in response to polyplexes formed at the effective N/P ratio of **S** (free polymer content of 0%), where free polymers do not exist, and polyplexes with free polymer content of 64%, 78%, and 86% (Figure 4A). We measured the cytokine expression of **S** polyplexes, untreated controls, unpackaged pDNA and commercial pDNA delivery reagents (jetPEI and LPF 2000). Twenty-four hours after administering polyplexes or controls, we performed bead-based cytometric assays to measure TNF-α and CCL-2 in macrophage-conditioned cell culture media (Figures S10–S11).

**Figure 4:**
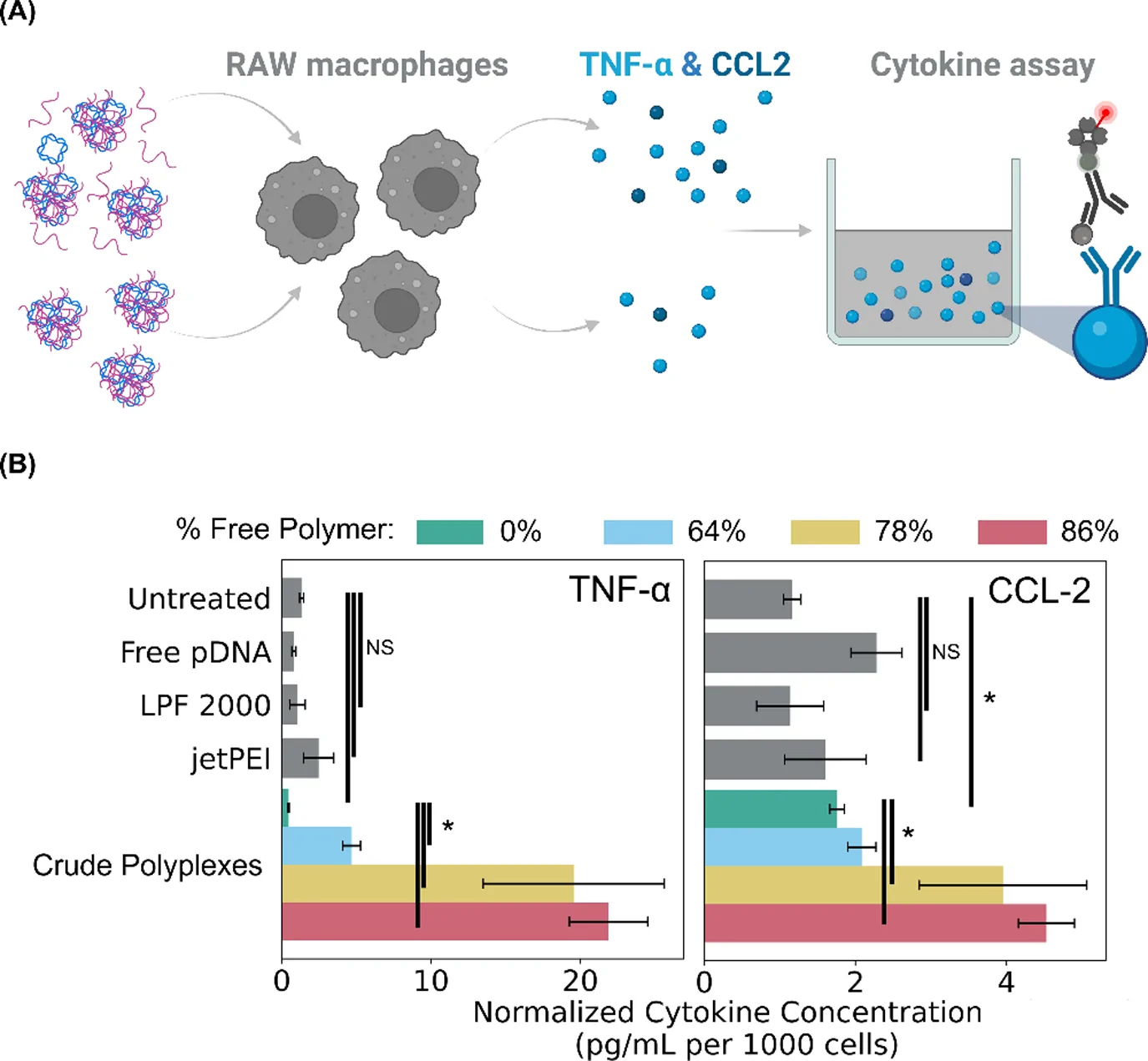
We measured the cytokine secretion in response to **S** polyplexes as a function of free polymer content. (A) We performed multiplexed cytometric bead assays to quantify the induction of TNF-α and CCL-2 and observed progressively higher cytokine expression with increasing free polymer content. (B) Polyplexes with 78% and 86% free polymers induced more than double the expression of TNF-α compared to polyplexes with 64% free polymers. We observed similar trends for CCL-2. Unpackaged pDNA, and commercial reagents jetPEI and LPF 2000, had similar cytokine levels as untreated cells.

TNF-α and CCL-2 secretion levels increased steeply with increasing free polymer content (Figure 4B). Interestingly, we did not observe appreciable changes in TNF-α and CCL-2 for jetPEI and LPF 2000, compared to untreated cells. In the previous section, we discussed how jetPEI polyplexes, which have a higher effective N/P ratio (2.5^39^ vs 1.08) and fewer free polymers than **S**, load similar amounts of pDNA as **S** while minimizing the mass ratio of toxic polycations. Clearly, these unique solution properties of jetPEI allow them to maintain baseline levels of cytokine expression, scarcely discernible from untreated cells. At the same N/P ratio of 5, jetPEI contains only 48% free polymer compared to 78% for **S**, but **S** elicits 8× higher TNF-α secretion than jetPEI (Figure 4B). Unlike **S**, polymers like jetPEI, that pack pDNA efficiently while minimizing free polymer content will promote efficient intracellular pDNA delivery without provoking inflammation.

**S** polyplexes formed at the effective N/P ratio of 1 (0% free polymers) did not elevate TNF-α and CCL-2 concentrations beyond baseline levels (untreated cells). However, **S** polyplexes containing 78% and 86% free polymers elicited more than ten times the TNF-α expression as untreated cells (Figure 4A). CCL-2 levels were similarly elevated for these two samples but differences were less pronounced. Cytokine levels were moderate for **S** polyplexes formed with slightly fewer free polymers (64%). This suggests a free polymer concentration threshold above which cytokine secretion levels spike dramatically. Our in vitro observations are consistent with in vivo reports by Sundaram et al., who found that increasing the amount of free polymers in PEI polyplexes resulted in a 6-fold increase in TNF-α in mouse models.^44^ Without any involvement from polyplexes whatsoever, free polycations may independently associate with cell membranes, disrupt cell membrane integrity, and activate toll-like receptors.^45,46^ They may also form membrane attack complexes.^37,47^ Therefore, the free polymer-dependent increase in TNF-α expression may be triggered by one or more of these inflammatory pathways. However, delineating the exact pathway responsible is out of the scope of this work and remains a direction for future research. After observing that free polymers spiked cytokine levels, we removed free polymers via AF4 prior to intracellular pDNA delivery, which we discuss next.

### Removing Free Polymers Reversibly Abolishes Polyplexes’ pDNA Delivery Capacity and Toxicity

We asked how removing free polymers via AF4 would alter the transgene expression and cytotoxicity of **S** polyplexes (Figure 5). Crude polyplexes were formed via pipet mixing with 78% free polymer; later AF4 removed free polymers to yield purified polyplexes devoid of free polymers. We tested whether controlled readdition of free polymers would restore transfection outcomes by adding back free polymer to purified polyplexes, thereby increasing the free polymer content from 0% (64%, 78%, and 86%). Overall, we compared crude polyplexes, AF4-purified polyplexes, and AF4-purified polyplexes spiked with free polymers (Figure 5A). Purified polyplex fractions were added to HEK293T cells within 4 h. If AF4-purified polyplexes are held beyond 6 h, the hydrodynamic size increases by 18% (Figures S13 and S14). To adjust for the dilution during AF4, it is important to determine the pDNA concentration of the AF4-purified polyplexes, which we quantified in Figure S12.

**Figure 5:**
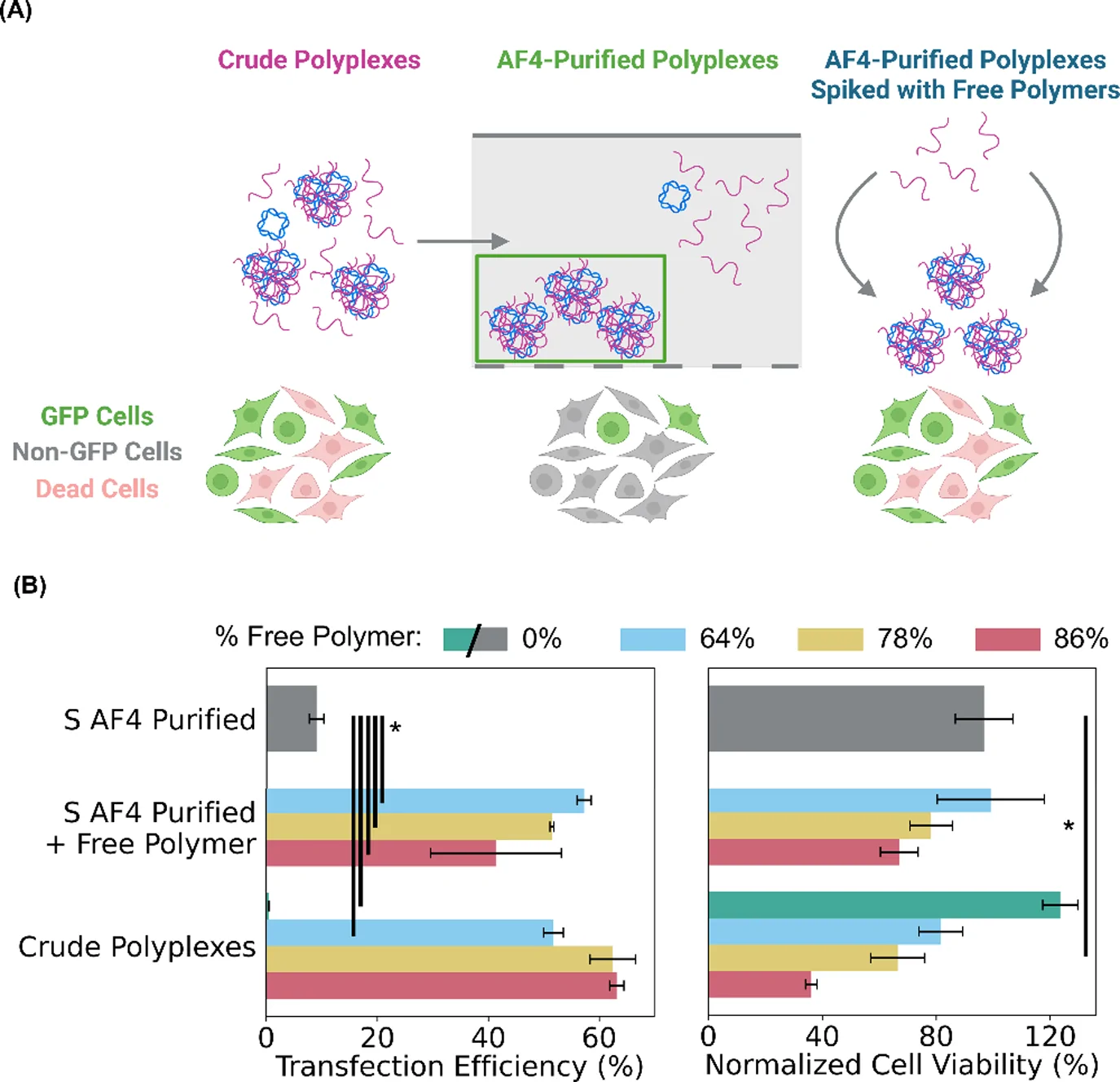
Free polymers, rather than polyplexes, control pDNA delivery outcomes. (A) We compared the transfection efficiency and cytotoxicity of crude polyplexes, AF4-purified polyplexes (where free polymers have been removed), and AF4-purified polyplexes spiked with free polymers. (B) AF4 depressed the transfection efficiency and proportionally increased the cell viability of **S** polyplexes. However, adding back free polymers to the AF4-purified polyplexes returned the transfection efficiency and toxicity to levels comparable to crude polyplexes.

After delivering GFP-encoding pDNA loaded in polyplexes, we measured GFP expression and cellular metabolic activity via flow cytometry and CCK-8 assays. LPF 2000 and jetPEI served as positive controls; untreated cells and unpackaged pDNA were negative controls (data for cell culture controls is furnished in Figure S15A). Crude polyplexes formed with 78% free polymer mediated GFP expression in 62% of cells while maintaining a cell viability of 66%. However, AF4-purified polyplexes recorded a 53% decrease in transfection efficiency (which fell from 62% to 9%) and 31% increase in cell viability (increased from 66% to 97%). Although free polymers assist polyplexes with endocytic uptake and endolysosomal processing, they also exert cytotoxic effects.^48,49^ When we added back free polymers removed during AF4 purification, we restored the pDNA delivery trends observed for crude polyplexes (Figure 5B). After clarifying the biological role of free polymers in **S**, we repeated this study for jetPEI (Figure S15B) and observed similar trends. Ogris and Wagner applied size exclusion chromatography (SEC) to separate free PEI from polyplexes.^38^ SEC-aided removal of free polymers did not impact cell membrane association and endocytosis but altered polyplex sizes due to column-induced aggregation and trapping of large species, limiting the capacity to properly compare crude polyplexes with SEC-purified polyplexes. Because AF4 employs an open channel without stationary phase packing, we deftly avoided these aggregation artifacts introduced by SEC and compared size-matched polyplexes from the crude and AF4-purified populations and quantified the contributions of free polymers to intracellular pDNA delivery.

## Limitations

Although AF4 is a unique analytical tool that yielded insights of outstanding fundamental importance in polymer-mediated pDNA delivery, we must reckon with methodological challenges that hinder AF4 as a preparative technique. We note that our sample recovery (75–90% polyplex mass recovery) is likely due to nonspecific adsorption to the AF4 membranes and tubings; consequently we suffer pDNA loss (60% pDNA mass recovery). Careful pDNA quantification of AF4-purified polyplexes is essential to ensure correct dosing. We also note that AF4 is currently not scalable (≈20 μg pDNA per run) and that the 6 h time window of usability of AF4-purified polyplexes will pose logistical challenges. To test whether free polymers and polyplexes are cointernalized by cells, we need to map the intracellular localization of free polymers and polyplexes via fluorescence imaging. Fluorescent labeling of polymers and pDNA molecules can also provide insight into pDNA loss during AF4 due to polyplex disassembly by using Förster Resonance Energy Transfer (FRET) spectroscopy. We encountered two methodological challenges in performing AF4 with fluorescent labeled polymers and pDNA: (1) hydrophobic fluorescent dye molecules cause pDNA/polymers to adsorb irreversibly and be lost to AF4 membranes, (2) we need light scattering detectors equipped with special filters that block fluorescence.

## Conclusion

Polymeric carriers of nucleic acids remain poised on the cusp of clinical success but researchers lack analytical tools to fully characterize polyplexes. Similar to how empty viral capsids and empty LNPs exert immunogenic and toxic effects during gene delivery, free polymers—excess polymers that exchange dynamically with pDNA-bound polymers–also dictate polyplexes’ therapeutic fate. To decipher how free polymers shape the physical properties and pDNA delivery performance, we nondestructively fractionated free polymers from purified polyplexes under laminar flow, leaving behind structurally intact polyplexes. By directly addressing the confounding influences of free polymers, we established that polymer composition, but not microstructure, governs polyplex solution properties. Finally, we elucidated how free polymers modify immunogenicity, toxicity, and pDNA delivery efficiency. To independently tune transgene expression, toxicity, and immunogenicity, we will combine AF4 with the readdition of free polymers conjugated with small-molecule inhibitors of toxicity and inflammation in future work.

Unlike centrifugation, AF4 yielded purified polyplexes with comparable sizes and morphologies as crude polyplexes. Thus, we removed free polymers while avoiding the polyplex rupture and aggregation triggered by centrifugal stresses. Eliminating the background light scattering contributions of free polymers dramatically altered our physical understanding of polyplex properties. Compared to crude polyplexes, which are dominated by free polymers, AF4-purified polyplexes were twice as large and loaded 2–4 × more pDNA. We observed that polymers like jetPEI, which have higher effective N/P ratios, formed polyplexes with a smaller ratio of free-to-bound polycations compared to polymers like **S**, **B**, and **G**, which had lower effective N/P ratios. Further, microstructure-dependent pDNA loading trends that we had earlier reported among **S**, **B**, and **G** vanished after AF4 purification. Structure–function relationships derived from crude polyplexes appear to be biased by free polymer contributions and do not accurately reflect the intrinsic properties of the polyplexes themselves. Given that N/P ratios as high as 5–100 are routinely reported, published polyplex solution properties, including our own previous work,^37^ must be revisited in the light of free polymers’ contributions. We propose that the effective N/P ratio should be reported for any new polymeric gene carriers in future publications.

Transfection efficiency was lower in AF4-purified polyplexes (≈ 10% vs ≈ 60%) albeit with improved cell viability (≈ 100% vs ≈ 70%) compared to crude polyplexes. Upon reintroducing free polymers, we restored the transfection efficiency to levels comparable to crude polyplexes. This suggests that free polymers, rather than polyplexes themselves, drive intracellular pDNA delivery. We may observe some differences in the case of backbone-degradable polycations, which are considered less toxic than **S**, **B**, **G**, and jetPEI; the removal of free polymers may not produce as drastic an improvement in cell viability for these classes of polymers. Our finding that free polymers govern delivery outcomes and convolute solution properties likely extends beyond polyplexes to lipopolyplexes, micelleplexes, bottleplexes, dendriplexes, and other macromolecular complexes.

## Experimental Section

Detailed synthesis procedures and analytical methods can be found in the SI. Briefly, we synthesized **S**, **B**, and **G** via RAFT polymerization. Polyplexes were formed by adding cationic polymers to pDNA to achieve a final pDNA concentration of 200 ng/μL. Polyplexes were further diluted prior to batch analysis (batch DLS and batch MALS). Undiluted crude polyplexes were injected into the AF4 and fractions were characterized online with MALS, DLS and dRI detectors. We performed cytometric bead assays of cell culture media collected from polyplex-treated RAW macrophages to assess cytokine secretion. We transfected HEK293T cells to compare the pDNA delivery efficiency and cytotoxicity of crude polyplexes, AF4-purified polyplexes, and AF4-purified polyplexes spiked with free polymers. Data are presented as mean ± standard deviation. At least three measurements were performed. Statistical analyses were performed using Python. Tukey’s honest significant difference (HSD) test was used to perform pairwise comparison of sample means. Statistical significance was defined as p-value less than 0.05.

## Supplementary Material



## Notes

Detailed experimental methods, polymer characterization data, AF4 mass recovery calculations, shear stress calculations, supplemental TEM data, supplemental DLS and SLS data, supplemental pDNA delivery and cytokine measurements, and raw flow cytometry data (PDF)

## #

Center for Life Sciences, Mahindra University, Hyderabad, Telangana 500043, India
