# Supplementary material for: Free Polymers Skew Polyplex Solution Properties and Confound the Interpretation of Structure–Function Relationships
Source: ACS Polym Au. 2026 Jun 15;6(5):1329–38. doi: 10.1021/acspolymersau.6c00080 (PMC13673885; doi:10.1021/acspolymersau.6c00080)
Supplement: Supplementary file 1 [file lg6c00080_si_001.pdf]

Supporting Information

Free polymers skew polyplex solution properties and confound the interpretation of structure-function relationships.

Jessica L. Lawson,[†] Kaitlin C. Lesco,[‡,¶] Ram Prasad Sekar,[§] Grant Wheeler,[‖] S. Kim Ratanathanawongs Williams,[*,¶,†,‖] and Ramya Kumar[*,§,†,‖]

†*Materials Science, Colorado School of Mines, Golden, CO 80401*

‡*Bioenergy Science and Technology Directorate, National Laboratory of the Rockies, Golden, CO 80401*

¶*Department of Chemistry, Colorado School of Mines, Golden, CO 80401*

§*Chemical and Biological Engineering, Colorado School of Mines, Golden, CO 80401*

‖*Quantitative Biosciences and Engineering, Colorado School of Mines, Golden, CO 80401*

E-mail: krwillia@mines.edu; ramyakumar@mines.edu

Contents

1 Experimental section **S-4**

1.1 Materials S-4

1.2 Polymer synthesis S-4

1.3 Polymer characterization S-5

1.4 Polyplex formation S-5

1.5 Batch dynamic light scattering S-6

1.6 Batch static light scattering S-6

1.7 Polyplex separation via asymmetrical flow field flow fractionation S-7

1.8 Polyplex separation via centrifugation S-8

1.9 Transmission electron microscopy S-8

1.10 pDNA concentration determination in AF4 fractions. S-8

1.11 Biological studies S-9

1.12 Cytokine concentration measurements. S-11

2 Characterization of polymers investigated in this study. **S-12**

3 Detailed AF4 schematic **S-13**

4 Centrifugation vs. AF4: shear stress calculations **S-14**

5 Centrifugal separation of polyplexes: size and pDNA loading measurements. **S-16**

6 TEM images **S-17**

7 Supplemental MALS data. **S-20**

8 Supplemental data from cytokine quantification. **S-22**

9 Quantifying pDNA loading in AF4-purified polyplexes. **S-24**

10 Stability studies of AF4-purified polyplexes. **S-25**

11 Supplemental pDNA delivery results **S-27**

12 Immunoassay flow cytometry gating schemes **S-28**

1 Experimental section

1.1 Materials

N,N-dimethylformamide (DMF) was purchased from Spectrum Chemicals (New Brunswick, NJ). The CTA 4-cyano-4-(((ethylthio)carbonothioyl)thio)pentanoic acid (CEP; 95%) was purchased from Ambeed (Buffalo Grove, IL). 4,4'-azobis(4-cyanovaleric acid) (V501; >98%), 2-(diisopropylamino)ethyl methacrylate (DIP; 97%, 100 ppm MEHQ) and 2-hydroxyethyl methacrylate (HEMA; ≥99%, ≤50 ppm MEHQ) were purchased from Sigma-Aldrich (St. Louis, MO). DIP and HEMA monomers were purified by passing through basic alumina (Sigma-Aldrich). All other chemicals were used as received. Plasmids (pZsgreen N-1, 4626 bp) were purchased from Aldevron (Fargo, ND). JetPEI was purchased from Polyplus (New York, NY). Lipofectamine 2000 (LPF 2000) was purchased from Thermo Fisher Scientific (Waltham, MA). Dulbecco's Modified Eagle Medium (DMEM), fetal bovine serum (FBS), phosphate-buffered saline (1 × PBS), trypsin-EDTA (0.25%)-phenol red, antibiotic-antimycotic solution (100 ×), Opti-MEM I (reduced serum media), FluoroBrite-DMEM, and Ultrapure DNase/RNase-free distilled water were procured from Thermo Fisher Scientific (Waltham, MA). Heparin sodium salt from procine mucosa was purchased from Millipore Sigma (catalog # H3393). Cytokine multiplex assay kits (LEGENDplex Multi-Analyte Flow Assay Kit) were purchased from Biolegend (San Diego, CA). Cell Counting Kit-8 (CCK-8) was purchased from Dojindo (Kyushu, Japan).

1.2 Polymer synthesis

All copolymers were synthesized via reversible addition fragmentation chain transfer (RAFT) polymerization in the Carousel 12 parallel synthesizer (Heidolph; Wood Dale, IL) as previously described[1].

1.3 Polymer characterization

All NMR spectra were recorded with a JEOL ECA 500 instrument (JEOL; Peabody, MA). ^{1}H-NMR samples were prepared by dissolving ≈10 mg of polymer in 0.7 ml D_2O and the spectra were recorded with 32 scans and a relaxation delay of 10 s. Molecular weight distributions were determined via size exclusion chromatography (Agilent; Santa Clara, CA) using an Optilab differential refractive index detector (dRI) and a DAWN multi-angle light-scattering detector (SEC-MALS, Wyatt Technology; Santa Barbara, CA) in a 100 mM Na_2SO_4, 1 v% acetic acid buffer. Three columns were used in series for polymer separation: CATSEC-100, CATSEC-300 and CATSEC-1000 (Eprogen; Downers Grove, IL). The number of DIP and HEMA repeat units for each polymer were thus calculated:

$$RU_{DIP} = \frac{n_{DIP}(M_n - M_{CTA})}{n_{DIP}M_{DIP} + (1 - n_{DIP})M_{HEMA}} \tag{1}$$

$$RU_{HEMA} = RU_{DIP}\frac{1 - n_{DIP}}{n_{DIP}} \tag{2}$$

where n_{DIP} is the molar percent of DIP in the copolymer determined from ^{1}H-NMR, M_n is the copolymer molecular weight determined from SEC-MALS, and M_{CTA}, M_{DIP} and M_{HEMA} are the molecular weights of the CTA, DIP and HEMA monomers, respectively.

1.4 Polyplex formation

Polymer stock solutions were prepared by dissolving the polymer in UltraPure water to obtain 15.15 nmol of protonatable amine groups per milliliter (nmol N/mL). Polymer stock solutions were filtered using a 0.22 μm hydrophilic filter. Polyplexes were prepared by adding diluted polymer stock (diluted with 10 mM $NaNO_3$ or MilliQ water to obtain targeted N/P ratio) to equal volumes of diluted pDNA stock (diluted with 10 mM $NaNO_3$ or MilliQ water to obtain final desired pDNA concentration). jetPEI was diluted according to the manufacturer's protocol for the desired N/P ratio and pDNA concentration. The solutions were gently pipette mixed and incubated at room temperature for 45 min prior to characterization.

1.5 Batch dynamic light scattering

DLS was performed using a Brookhaven NanoBrook 90Plus (Brookhaven Instruments; Holtsville, NY). Polyplexes were prepared at a pDNA concentration of 200 ng/μL then further diluted with 10 mM $NaNO_3$ to 20 ng/μL for **S** and **G** and diluted with MilliQ water for **B** and jetPEI. Three DLS measurements were averaged for the final reported values. R_h values were calculated using the CONTIN regularization method, since this is suitable for particles with broad distributions.

1.6 Batch static light scattering

The polyplex molecular weight and radius of gyration (R_g) were determined via SLS using a Wyatt DAWN 18-angle, light scattering detector (Wyatt Technology; Santa Barbara, CA). The polyplexes were prepared in 10 mM $NaNO_3$ or MilliQ water at an N/P ratio of 5 and a pDNA concentration of 200 ng/μL. The polyplexes were diluted to different pDNA concentration between 5-15 ng/μL. Each sample was injected at a flow rate of 0.1 mL/min for 5-10 min until a stable signal was obtained from the light scattering detectors. The polyplex dn/dc values, which are necessary for accurate SLS measurements, were calculated from a weight average of the polymer dn/dc and the pDNA dn/dc, obtained using Wyatt Astra 8.2.0 software. Debye plots were generated using Wyatt Astra 8 software using a second order angular dependence. This model lends itself well to particles that are ≈100nm, since there is typically an exponential angular dependence for larger particles. Hu et al.[2] showed that polyplex molecular weight can be correlated to a DNA loading using the following equations:

$$m_{bound} = \frac{N/P_{eff}}{5} \times \frac{C_{polymer}}{C_{pDNA}} \times M_{pDNA} \tag{3}$$

$$\bar{N} = \frac{\bar{M}_{polyplex}}{m_{bound} + M_{pDNA}} \tag{4}$$

where m_{bound} is the mass of pDNA-bound polymer, N/P_{eff} are the effective N/P values (or the N/P of polymer to pDNA in the polyplexes excluding free polymers, approximated as 1 for **S**, **B**,

and **G** and 3 for jetPEI) furnished in Figure S7, $C_{polymer}$ and C_{pDNA} are the polymer and pDNA concentrations used in the polyplex formation, M_{pDNA} is the molecular weight of the pDNA and $\bar{M}_{polyplex}$ is the molar mass of the polyplex determined from SLS.

1.7 Polyplex separation via asymmetrical flow field flow fractionation

Fractionation was conducted using a TF2000 AF4 system (PostNova Analytics; Salt Lake City, UT) coupled with a DAWN HELEOS II multi-angle light scattering (MALS) with an integrated dynamic light scattering detector (DLS) and a T-rEX differential refractive index (dRI) detector (Wyatt Technology; Santa Barbara, CA). Polyplexes were prepared at an N/P ratio of 5 and a pDNA concentration of 200 ng/µL in 10 mM $NaNO_3$ or MilliQ water. A low salt concentration is ideal for AF4 separation as this prevents interactions with the channel membrane. We saw salt-induced aggregation with **B** and jetPEI samples, requiring us to switch to MilliQ water for these two samples.

Polyplex samples were injected without further dilution using a 100 µL injection loop. The AF4 channel was equipped with a 10 kDa MWCO modified regenerated cellulose membrane (PostNova Analytics; Salt Lake City, UT), a 350 µm wide Mylar spacer (27.5 cm tip-to-outlet length, maximum width of 2 cm narrowing to 0.5 cm). Carrier fluids were filtered using a 0.1 µm inline filter (Merck Millipore Ltd; Ireland). The sample was focused for 5 min at a crossflow of 3 mL/min and an inlet channel flow of 0.3 mL/min. After the focus step, the crossflow decreased exponentially (exponent 0.1) to 0 mL/min over 6 min. The crossflow remained at 0 mL/min for the remainder of the 30 min run to ensure complete elution of the polyplexes. The outlet flow was set to 0.4 mL/min. For the mobile phase, 10 mM $NaNO_3$ was used for **S** and **G**. Purified water was used for the analysis of jetPEI and **B**. For analysis, M_w, R_g, and R_h were calculated with Wyatt Astra 8 software using a second order Debye model since the polyplexes are larger 100 nm in diameter. Mass recovery was ≈75% (Figure S6 in the SI), calculated by dividing the area under the curve of the dRI concentration detector with and without an applied crossflow.

1.8 Polyplex separation via centrifugation

Polyplexes (1 mL) were prepared at an N/P ratio of 5 and a pDNA concentration of 50 ng/μL. Polyplexes were centrifuged in a VWR Micro 18R centrifuge (Radnor, PA) at 14K, 16.5K, and 19.5K RCF for 15 min at 4°C. The supernatant was collected, and the pellet was diluted back to 1 mL with MilliQ water. DLS and PicoGreen measurements were performed on both the pellet and the supernatant. TEM samples were prepared from the resuspended pellet.

1.9 Transmission electron microscopy

Polyplex morphology was visualized using a FEI Talos F200X TEM (Thermo Fisher Scientific, Waltham, MA), operated at 200 kV and equipped with a field emission gun (FEG). Polyplexes were collected after AF4-fractionation and centrifugation. The polyplex samples (2 μL) were deposited on copper coated formvar grids (200 mesh) and incubated for 1 min. This step was repeated twice for AF4 samples due to the high dilution in the AF4 channel. The grids were washed in UltraPure water four times and dipped in 2% uranyl acetate for 30 s. To remove the excess stain, grids were washed four more times with UltraPure water. The grids were then dried in vacuum desiccator overnight. After imaging, micrographs were processed with ImageJ to determine polyplexes sizes. The polyplex radii of 20-40 individual polyplexes were measured using ImageJ. TEM images and histograms of polyplex size as determined by TEM are furnished in Figures S4, S5 and S6 in the SI.

1.10 pDNA concentration determination in AF4 fractions.

We quantified the concentration of pDNA in the purified polyplex solution after AF4 for accurate pDNA dosing to cells. We created a standard plot of fluorescence versus pDNA concentration in our polyplexes. First, polyplexes were formed at a pDNA concentration of 200 ng/μL in MilliQ water and incubated for 45 min at room temperature. After incubation, we diluted the polyplexes 100× with MilliQ water to achieve a pDNA concentration of 2 ng/μL. The polyplexes were further diluted to concentrations between 0.1 and 2 ng/μL. We added equal volumes of a heparin solution

(20 mg/mL in MilliQ water) and incubated for 45 min. Heparin is a highly anionic molecule that competitively displaces pDNA from polyplexes, allowing us to quantify the total amount of pDNA in solution rather than just the unbound pDNA.[3,4]

After incubating with heparin, a PicoGreen assay was performed. To a black-bottom 96-well plate, 50 µL of sample was added in triplicate. PicoGreen was diluted according to the manufacturer's protocol (200×) with MilliQ water. Equal volumes of PicoGreen dilution was added to each well containing a sample and incubated for 5 min at room temperature. Heparin alone and polyplexes without heparin were used as controls. Fluorescence was measured in a microplate reader (Synergy H1, BioTek; Winooski, VT) with monochromator settings of excitation/emission 485/520 nm. The three sample measurements were averaged and used to create a standard curve (Figure S12).

To determine the pDNA concentration in the polyplexes after AF4 separation, we followed a similar protocol. The polyplexes were further diluted 10× to ensure a concentration in range of our standard curve. The polyplexes were mixed with equal volumes of heparin solution and incubated for 45 min. The PicoGreen assay was performed as previously mentioned. The standard curve was used to calculate the pDNA concentration after AF4.

We additionally performed stability studies on the AF4-purified polyplexes to ensure they were not aggregating prior to transfection. To do this we measured the R_h of the AF4-purified polyplexes compared to a crude polyplex control. The purified polyplexes were stable over the course of 7 h, which is enough time for our transfection experiments (Figures S13 and S14 in the SI). However, they aggregated severely if held for several days.

1.11 Biological studies

Cell culture

Human embryonic kidney cells (HEK293T) purchased from the American Type Culture Collection (ATCC, Manassas, VA) were grown in Dulbecco's modified Eagle medium (DMEM) supplemented with 10% fetal bovine serum (FBS) and 1% antibiotic-antimycotic using T-75 tissue culture flasks.

When cells reached approximately 80% confluency, the cells were passaged into fresh tissue culture flasks. The cell confluency and morphology were monitored on a regular basis using an inverted light microscope and the EVOS XL Core Imaging System (Thermo Fisher Scientific; Waltham, MA).

pDNA delivery

HEK293T cells were seeded in 24-well tissue culture plates at a density of 50,000 cells per well and incubated for 24 h at 37°C and 5% CO_2. After 24 h incubation, we performed transfection using purified polyplex fractions from AF4. The AF4-purified polyplexes had an effective N/P ratio of 1 for S and 2.5 for jetPEI polyplexes. Therefore, we spiked in low volumes of polymer stock to reintroduce free polymers into polyplex solutions and achieve the desired free polymer content. We diluted the crude polyplexes and controls to the same pDNA concentration as the AF4-purified polyplexes (Figure S12 in the SI). The positive controls (jetPEI and LPF 2000) were prepared following the manufacturers' protocol.

Before performing the transfection, we diluted the polyplexes with Opti-MEM (reduced serum media) at a ratio of 1:2 to achieve a pDNA concentration of 1 μg per well. The diluted polyplexes were added to the cells and incubated for 4 h at 37°C and 5% CO_2. Then, DMEM (1 mL) was added to each well, and transfected plates were transferred back to the incubator. After 24 h incubation, the media was replaced with fresh DMEM (1 mL) and incubated for an additional 24 h. The cells were trypsinized and centrifuged for 5 min at 1000 rpm to sediment the cell pellets. Then cells were washed with 1 $\times$PBS and resuspended in a 1 $\times$PBS solution containing 400 nM Calcein Red viability dye and 2% FBS. The cells were filtered using cell strainer capped FACS tubes (5 mL). GFP fluorescence was measured using spectral flow cytometry (Cytek Northern Lights, Fremont, CA) with a 488 nm excitation laser line with autofluorescence correction. The flow cytometry raw data was analyzed using FCS Express, and the percentage of GFP expression was calculated.

N/P ratios for cell culture were converted to % free polymer as follows:

N/P	S	jetPEI
1	0%	0%
3	64%	14%
5	78%	48%
7.5	86%	66%
N/P_{eff}	1.08	2.58

Cell viability measurements

The cell viability of polyplexes was measured after 48 h incubation via CCK-8 (Cell Counting Kit-8) assay. The stock CCK-8 solution was diluted with FluoroBrite (with 10% FBS). Cell culture media was aspirated carefully without disturbing cell monolayers, and diluted CCK-8 solution was added to the cells. Then cells were incubated at 37°C, 5% CO_2 and 90-95% humidity for 4 h. After 4 h of incubation, 100 L of the supernatant was transferred into 96-well clear bottom plates. The absorbance was measured at 460 and 720 nm using a microplate reader (Synergy H1, BioTek, Winooski, VT). To correct for background noise/air bubbles, the absorbance values at 720 nm were subtracted from those measured at 460 nm. Cell viability was calculated and compared with untreated and control samples.

1.12 Cytokine concentration measurements.

We measured cytokine secretion in the cell culture supernatant of macrophages using a cytometric bead assay, the LEGENDplex Multi-Analyte Flow Assay Kit (Biolegend; San Diego, CA). Briefly, RAW 264.7 cells were seeded in 24-well plates with a cell density of 2×10^5 cells and incubated for 24 h at 37°C, 5% CO_2, and 90-95% humidity. Polyplexes were formulated at various free polymer percents and diluted using Opti-MEM to achieve a pDNA concentration of 1 µg per well for transfection. pDNA delivery was carried out as previously described. 24 h after transfection, the cell culture supernatant was removed carefully and centrifuged at 10K RPM for 5 min to remove debris. We examined two different cytokine responses (TNF-α, CCl-2). The cytokine assay was performed with collected media following the Biolegend manufacturer instructions. Simultaneously, CCK-8 solution was added to the remaining cells, incubated for 3 h, and cell

viability was measured at 460 nm. Gating schemes are furnished in Figure S16.

2 Characterization of polymers investigated in this study.

Polymer	p(DIP$_n$-*co*-HEMA$_m$) n	m	M_n (kDa)[a]	Đ[a]	mol% DIP[b]	dn/dc[c]
**S**	45	43	15.4	1.11	51%	0.1467
**B**	46	41	15.3	1.25	53%	0.1528
**G**	42	47	15.4	1.07	47%	0.1586
**jetPEI**	-	-	-	-	-	0.1520[d]

Figure S1. Polymers were characterized via SEC-MALS and ^{1}H-NMR to determine molecular weight and composition, respectively. The polymer dn/dc values were calculated experimentally through batch injections into the dRI detector at known concentrations. The polymer dn/dc values were used to calculate the polyplex dn/dc from the weight average of the polymer dn/dc and pDNA dn/dc. This value is critical for MALS measurements. [a]From SEC-MALS, [b]from ^{1}H-NMR, [c]from batch dRI measurements. [d]Since jetPEI is an expensive commercial reagent, we had insufficient material to obtain the dn/dc directly. Therefore, the dn/dc for a linear 25 kDa PEI (polyethyleneimine) sample was used to approximate the dn/dc for jetPEI.

3 Detailed AF4 schematic

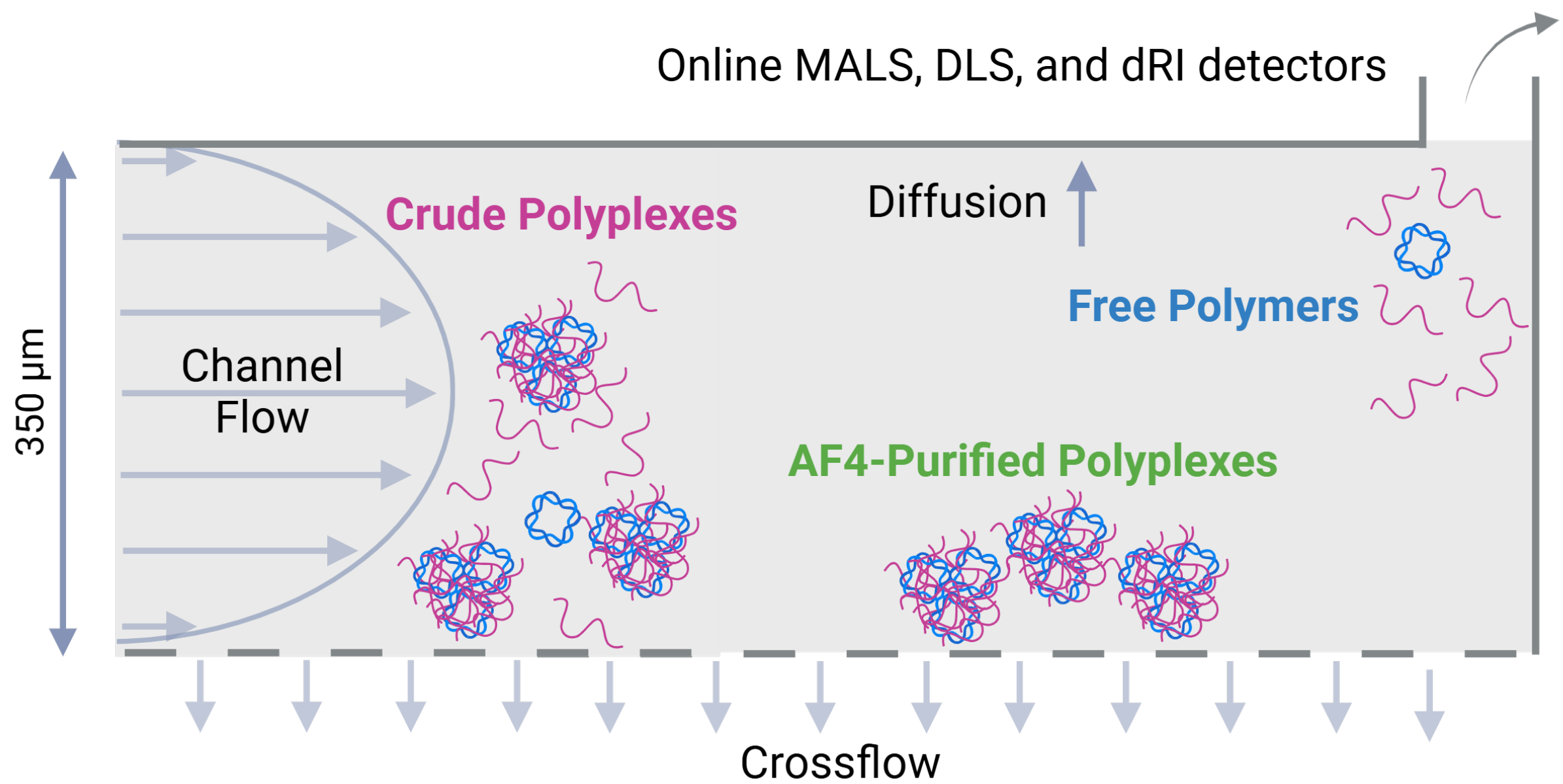

Figure S2. Asymmetrical flow field-flow fractionation (AF4) operates under a laminar flow regime. During AF4, a crossflow is applied to force the sample towards the semi-permeable membrane. Fast diffusing, small species such as free polymers counteract the crossflow and, on average, occupy the center of the channel. Slower diffusing, larger species such as polyplexes remain near the semi-permeable membrane. Because the channel flow adopts a parabolic velocity profile characteristic of laminar flow regimes, smaller species that occupy regions of faster flow will elute faster than larger species, resulting in shorter retention times into online multi-angle light scattering (MALS), dynamic light scattering (DLS), and differential refractive index (dRI) detectors.

4 Centrifugation vs. AF4: shear stress calculations

First, we calculated the Reynolds' number for the AF4 channel to determine the flow regime:

$$R_e = \frac{\rho v H}{\mu} = 0.33 \tag{5}$$

where ρ is the density of the mobile phase (1000 kg/m^3 for water), H is the height of the AF4 channel (350 μm), μ is the viscosity of the mobile phase (10^{-3} Pa sec for water at 20°C) and v is the velocity of the mobile phase calculated as follows:

$$v = \frac{Q}{A} = 9.52 \times 10^{-4} m/sec \tag{6}$$

where Q is the volumetric flow rate (0.4 mL/min for our experiments) and A is the cross sectional area of the AF4 channel (from 2 cm maximum channel width, W, 350 μm channel height, H). This small Reynold's number indicates that AF4 operates in the laminar flow regime. Next, we calculated the shear stress at the wall of the AF4 channel:

$$\tau_{wall} = \frac{6\mu Q}{H^2 W} = 0.016 Pa \tag{7}$$

For the determination of the shear stresses in centrifugation, we centrifuged our samples at 12K RPM and our centrifuge has a radius of ≈10 cm. We determine the velocity, v for centrifugation as follows:

$$v = \frac{2\pi N R}{60} = 120 m/sec \tag{8}$$

where N is the rotational speed of the centrifuge in RPM and R is the radius of the centrifuge. We calculated the Reynold's number for centrifugation:

$$R_e = \frac{\rho v H}{\mu} = 2.4 \times 10^6 \tag{9}$$

This large Reynold's number indicates centrifugation operates in the turbulent flow regime. Therefore, we must consider the frictional forces during centrifugation (Darcy's friction factor, f). The equation for the shear stress in centrifugation becomes:

$$\tau_{wall} = \frac{f \rho v^2}{8} = 36kPa \tag{10}$$

In summary:

	**Fluid Velocity v (m/sec)**	**Reynolds' Number (R_e)**	**τ_{wall} (Pa)**
**AF4**	9.52×10^{-4}	3.30×10^{-1}	1.6×10^{-2}
**Centrifuge**	1.20×10^{2}	2.4×10^{6}	3.6×10^{4}

5 Centrifugal separation of polyplexes: size and pDNA loading measurements.

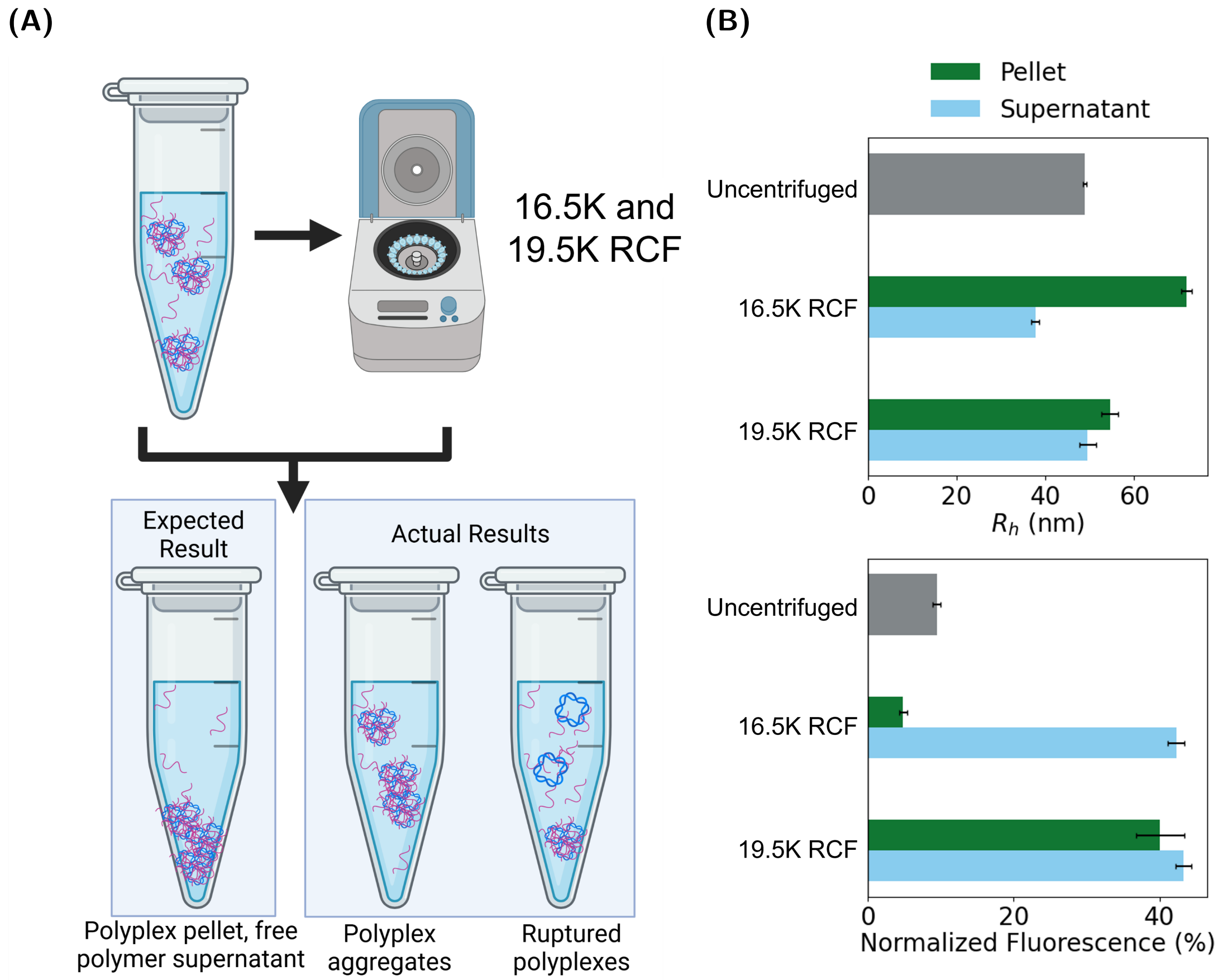

Figure S3. Centrifugal separation of free polymers from crude polyplexes disrupts polyplex stability at high centrifugal forces (19.5K RCF) and induces aggregation at low centrifugal forces (16.5K RCF). (A) We tested two centrifugation conditions (16.5K and 19.5K RCF) close to the maximum operating limit of a lab centrifuge. (B) We compared polyplex hydrodynamic size (DLS) and pDNA encapsulation (via dye exclusion) of uncentrifuged crude polyplex controls, and the pellet and supernatant from the two centrifugation conditions tested. Control polyplexes (not centrifuged) exhibited radii around 60 nm and less than 10% fluorescence, indicating efficient pDNA encapsulation. However, at the lower RCF, we triggered polyplex aggregation in the pellet. At the higher RCF, we lost most pDNA to the supernatant and polymers were no longer bound to pDNA in the pelleted polyplexes. Created with Biorender.com.

6 TEM images

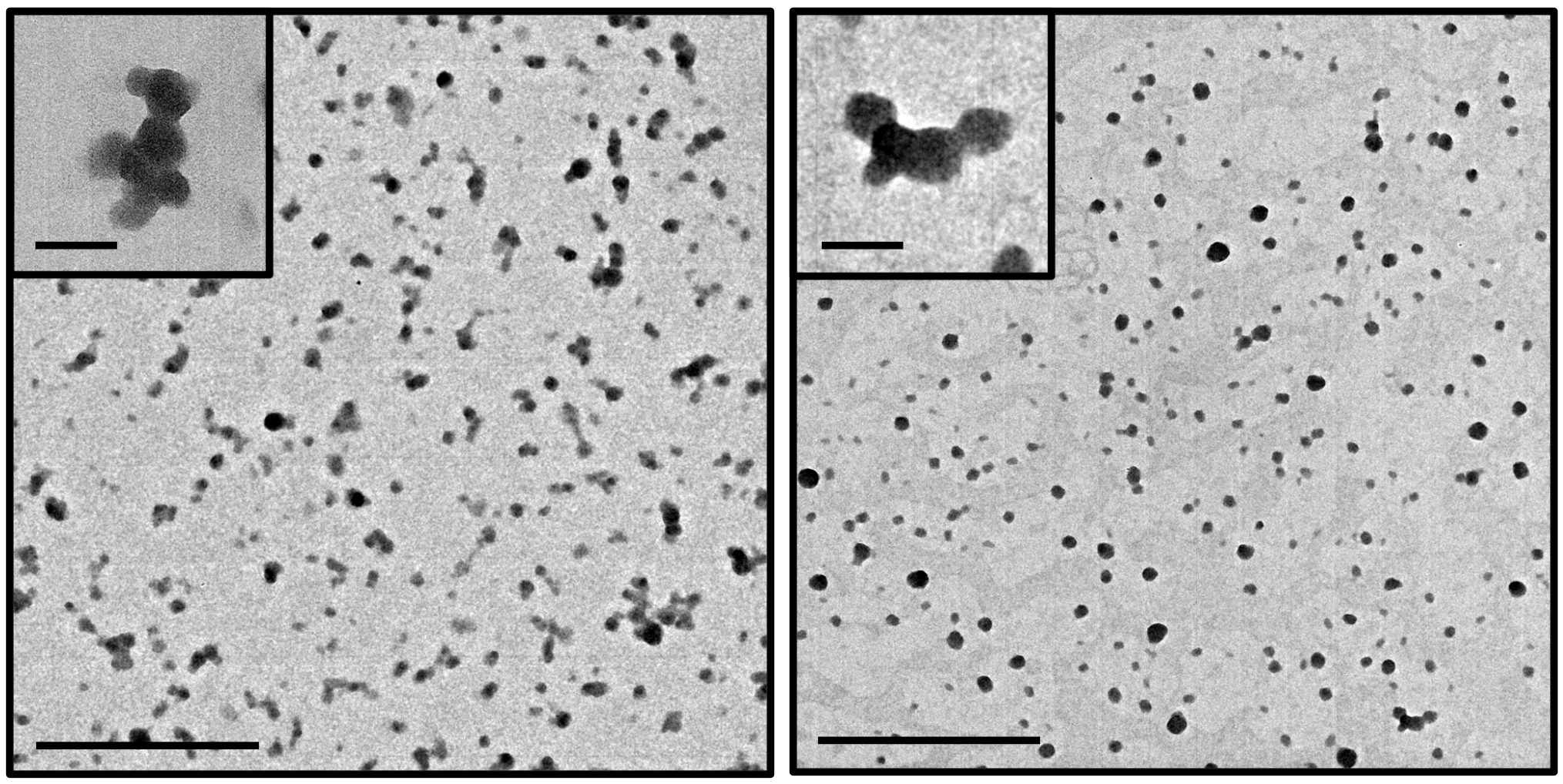

Figure S4. (A) & (B) TEM images of polyplexes that underwent centrifugation at different speeds. TEM captured changes in polyplex morphology and size relative to pre-centrifugation polyplexes (size quantification in Figure S6). Scale bars are 1 μm and 100 nm for the outset and inset, respectively.

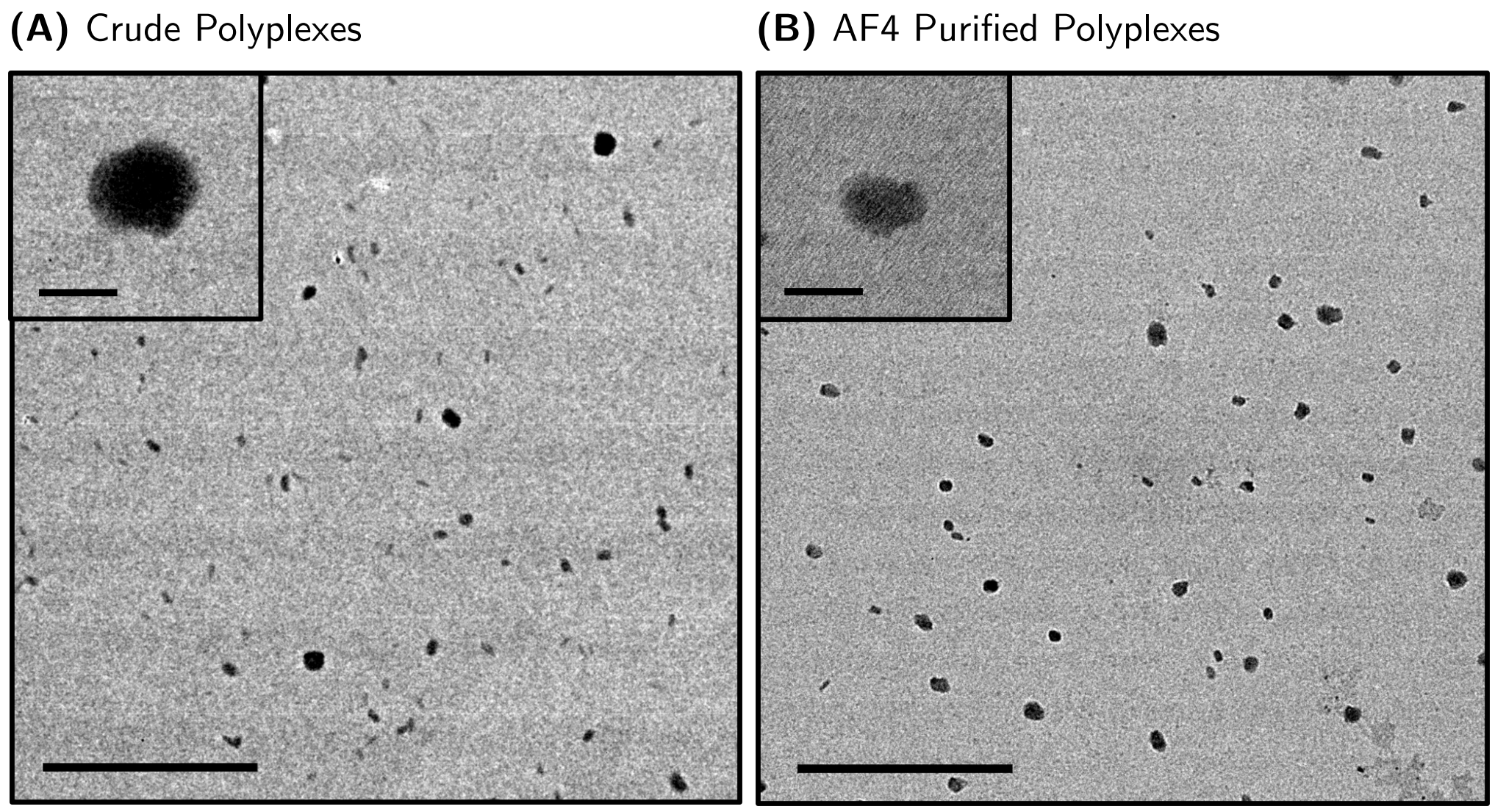

Figure S5. TEM images for (A) crude and (B) AF4 purified polyplexes were used to observe any changes in polyplex morphology and size due to AF4 purification. ImageJ was used to measure polyplex sizes to plot histograms of the polyplex size distribution in Figure S6. Scale bars are 1 μm and 100 nm for the outset and inset, respectively.

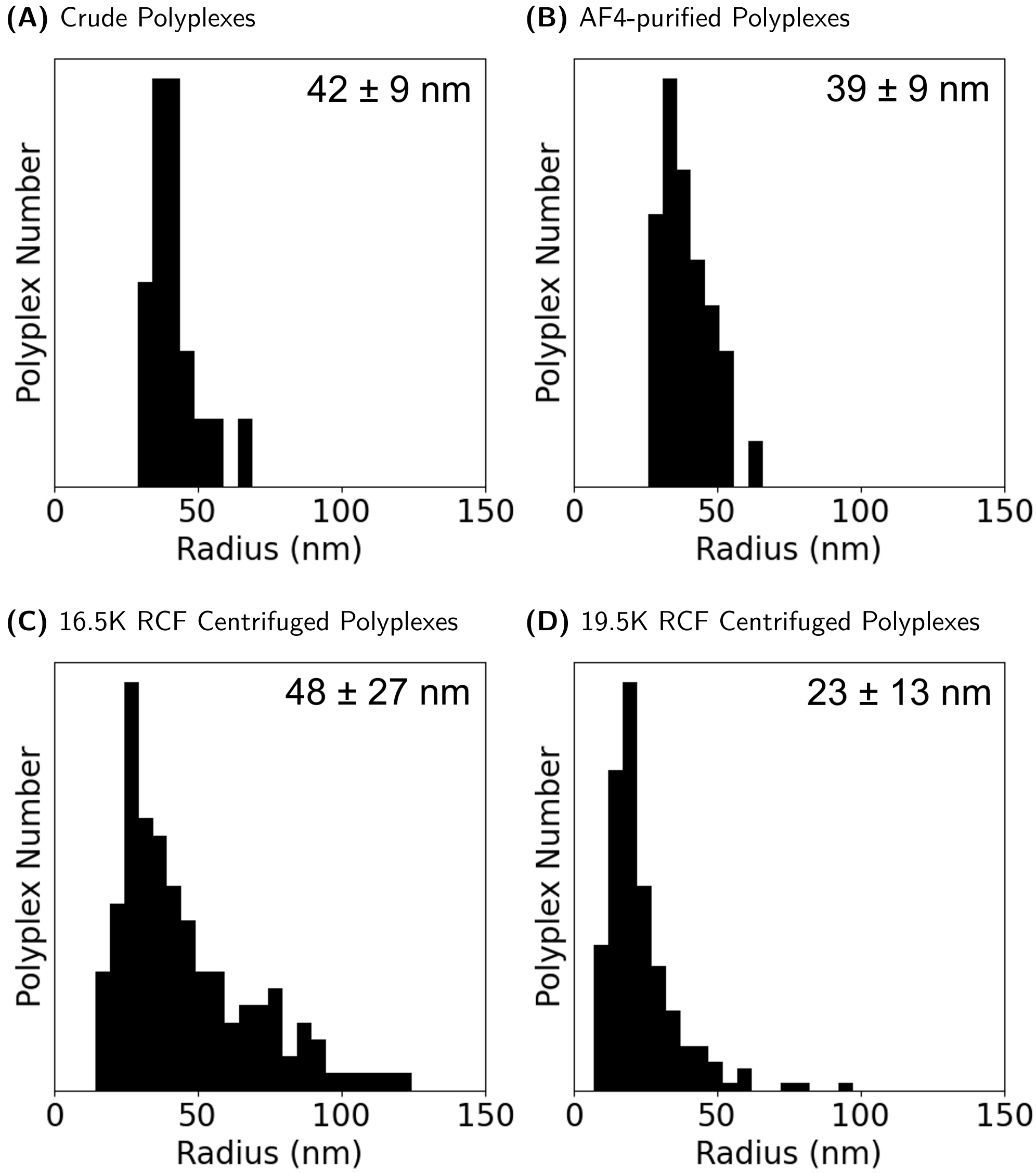

Figure S6. Using ImageJ, we individually measured the radii of each polyplex in TEM images. These histograms represent polyplex sizes measured from TEM images and particle counts (n). (A) Size distribution of crude **S** polyplexes (n=20) from TEM. (B) For AF4-purified polyplexes (n=39), their morphology, and size distribution remained unchanged compared to crude polyplexes. (C) & (D) After centrifugation at different speeds, crude **S** polyplexes either aggregated or ruptured. In (C), polyplexes displayed a broader size distribution and larger polyplex radii (n=148), suggesting aggregation. In (D), **S** polyplexes (n=201) assumed smaller polyplex radii, suggesting rupture.

7 Supplemental MALS data.

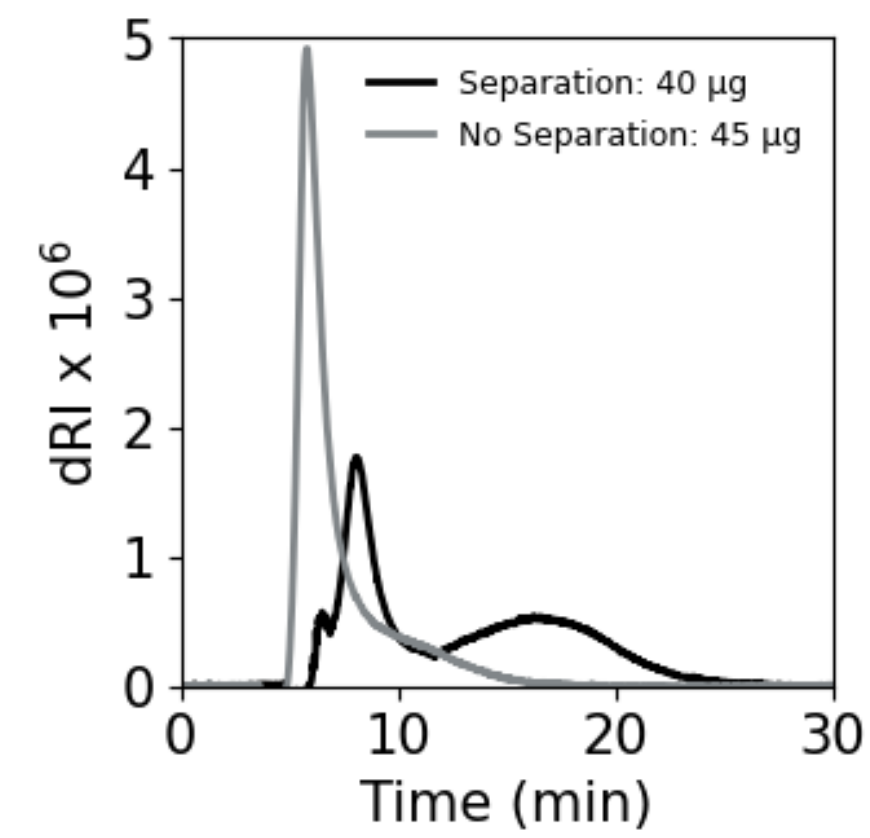

Figure S7. To determine the mass recovery after AF4, the total eluted mass from separated AF4 fractions (cross-flow was applied to separate free polymers from polymers) was compared to the total eluted mass from a control experiment where separation did not occur (no cross-flow applied). The dRI signal for the separated and unseparated samples are shown here. The estimated mass recovery for our AF4 experiments was 75-90%.

Polyplex	R_g (nm)	M_W (MDa)	N/P_{eff} MB	N/P_{eff} Lit	A_2 (mol mL/g^2)
**S**	104 ± 2	5.9 ± 1.1	1.08 ± 0.18	0.68	1.96×10^{-5}
**B**	91 ± 4	6.7 ± 3.1	1.88 ± 0.09	0.91	1.00×10^{-6}
**G**	98 ± 4	4.7 ± 0.9	1.36 ± 0.21	0.88	-1.40×10^{-5}
**JetPEI**	101 ± 5	22.7 ± 18.8	2.58 ± 0.15	2.50–3.00	3.11×10^{-5}

Figure S8. Tabulated R_g and M_W results for the first fraction (small molecules) from AF4-MALS. In general, the R_g and M_W were similar to uncomplexed pDNA, which scatters light more strongly than free polymers. We expect this peak is composed of a mixture of unbound pDNA and polymers and small, partially condensed polyplexes. We also provide the second virial coefficient (A_2) from batch MALS. A_2 was very small for all polyplexes; polyplex–solvent interactions did vary much across the four groups. We also compared the N/P_{eff} from AF4 mass balance (MB, first column) and estimates from previous studies by Lawson et al.[1] and Hu et al.[2] in the second column (Lit)

Here, we assumed the "free polymers" peak in the first eluted fraction is comprised solely of free polymers and that mass loss is comparable across all populations (free polymers, free pDNA, polyplexes).

$$N/P_{eff} = 5 - N/P_{free} \tag{11}$$

$$N/P_{free} = \frac{M_{FP}}{M_P} \times 5 \tag{12}$$

$$N/P_{free} = \frac{M_{Frac1}}{(M_{Frac1} + M_{Frac2}) \times \%P} \times 5 \tag{13}$$

Where N/P_{eff} is the effective N/P ratio of the polyplexes, N/P_{free} is the amount of free polymers in terms of N/P ratio, M_{FP} is the mass of free polymer, M_P is the total mass of polymer, M_{Fracx} is the mass of the x fraction from AF4, and $\%P$ is the mass fraction of polymer in the original polyplex formulation. The $\times$ 5 term comes from the N/P ratio of 5 at which we formed polyplexes.

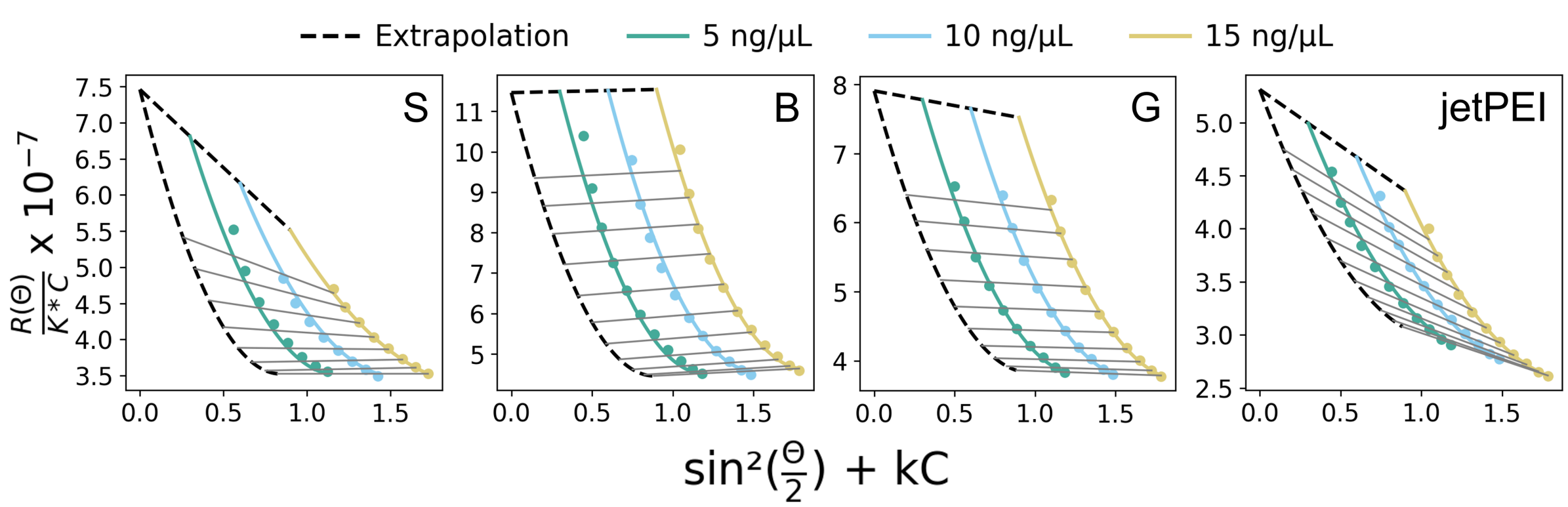

Figure S9. Representative Debye plots for **S**, **B**, **G**, and jetPEI from batch MALS estimated polyplex M_w, R_g, and pDNA molecules loaded per polyplex.

8 Supplemental data from cytokine quantification.

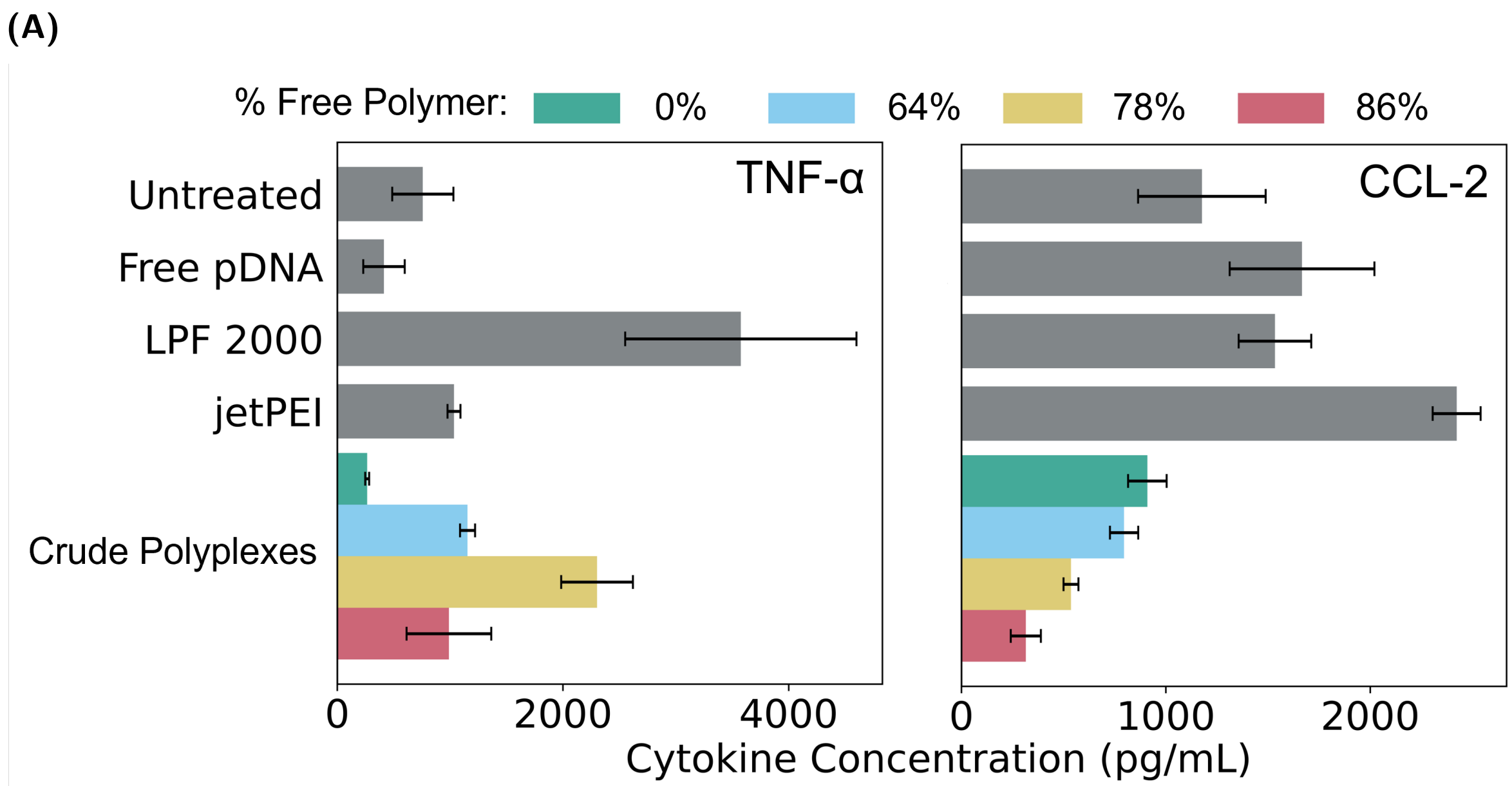

Figure S10. Cytokine secretion levels for different sample groups. Cytokine concentrations are not normalized to cell numbers in this plot.

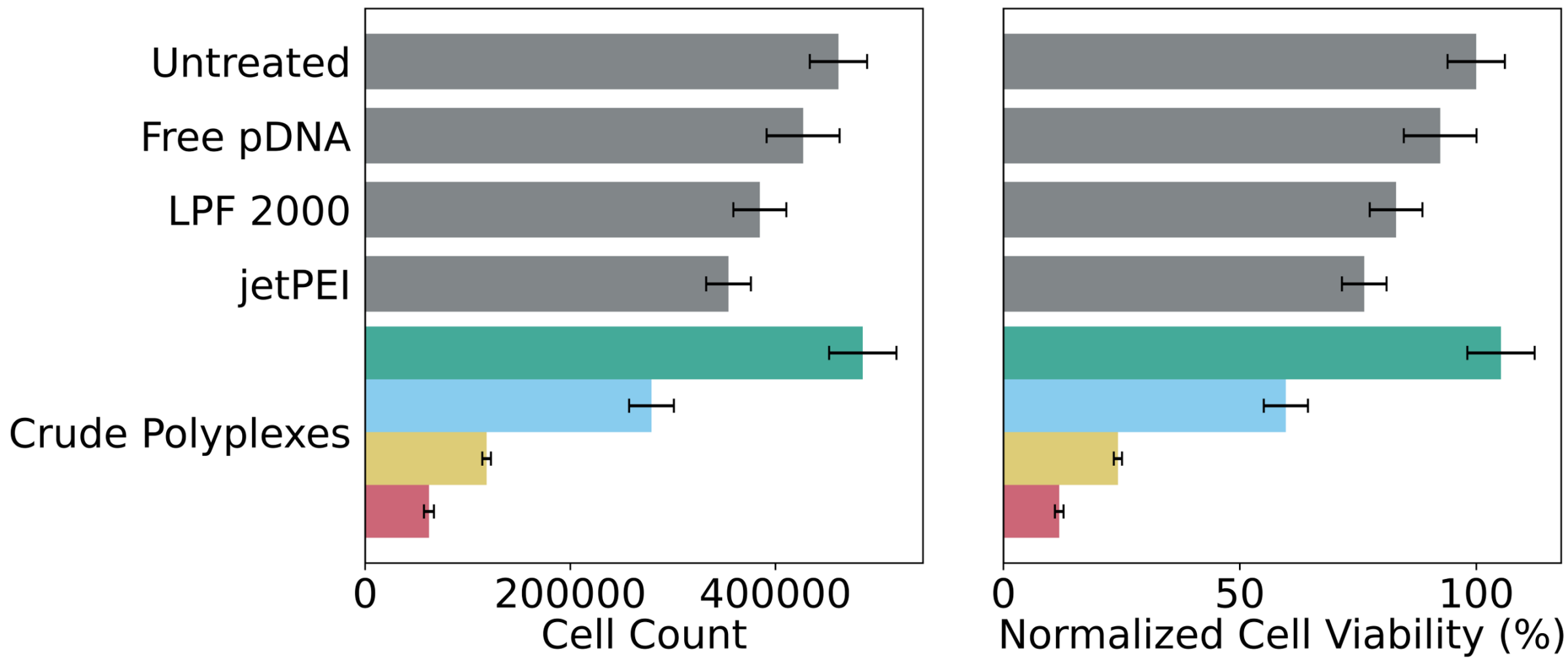

Figure S11. RAW 264.7 macrophages showed characteristic N/P-dependent cytotoxicity. To avoid confounding effects caused by large differences in toxicity levels, we calculated cell numbers for each treatment group from CCK-8 assays. These cell numbers were used to transform cytokine concentrations in Figure S10 to a per cell basis in Figure 4 of the main manuscript.

9 Quantifying pDNA loading in AF4-purified polyplexes.

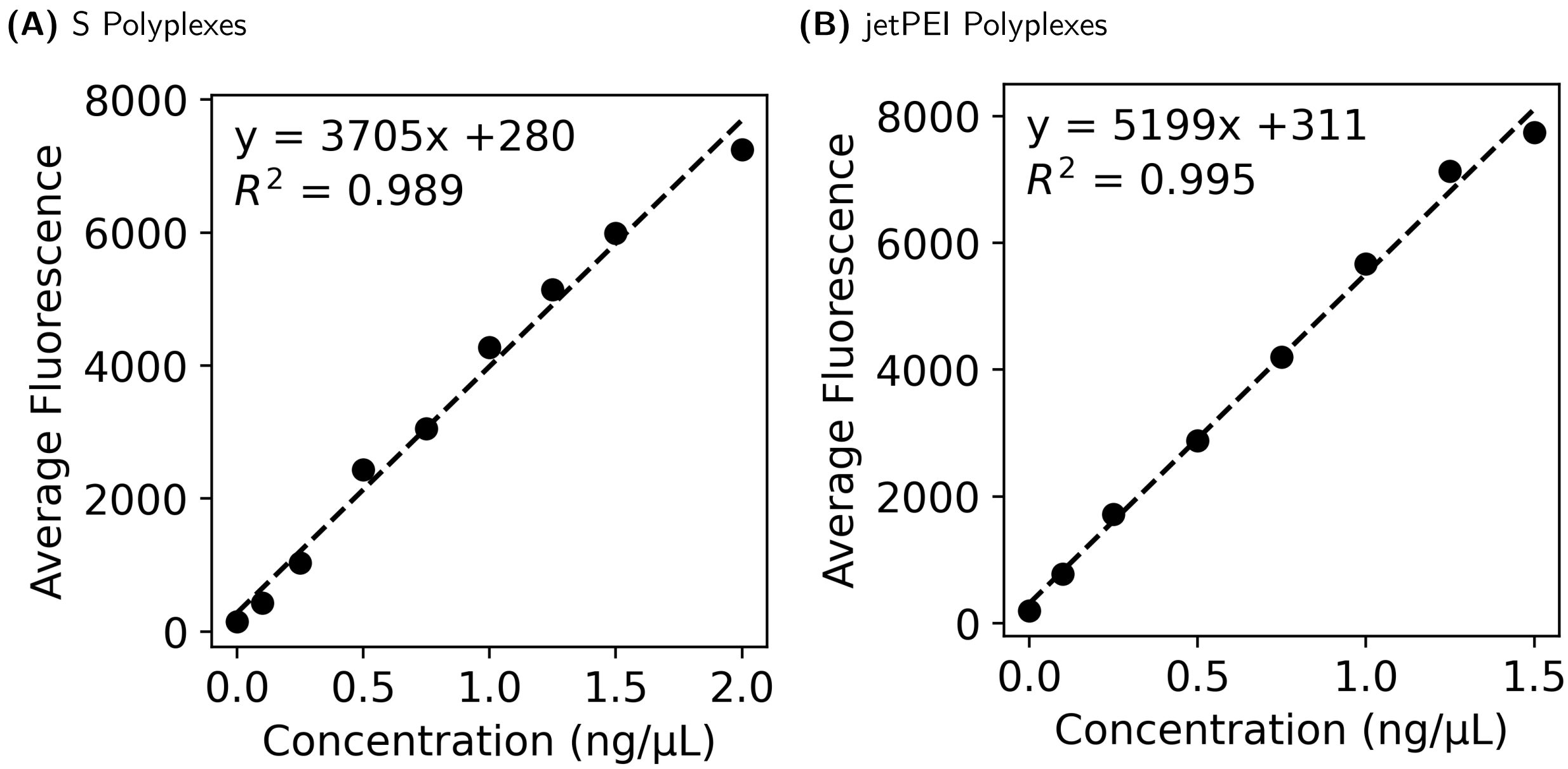

Figure S12. We determined the concentration of pDNA in purified polyplexes obtained after AF4-aided removal of free polymers. Calibration plots were constructed by performing PicoGreen assays. We used heparin to completely liberate pDNA in polyplexes to measure the total pDNA concentration rather than just unbound pDNA. We measured PicoGreen fluorescence at various polyplex concentrations. pDNA concentration estimates for (A) **S** polyplexes and (B) jetPEI polyplexes, both after AF4, are shown.

10 Stability studies of AF4-purified polyplexes.

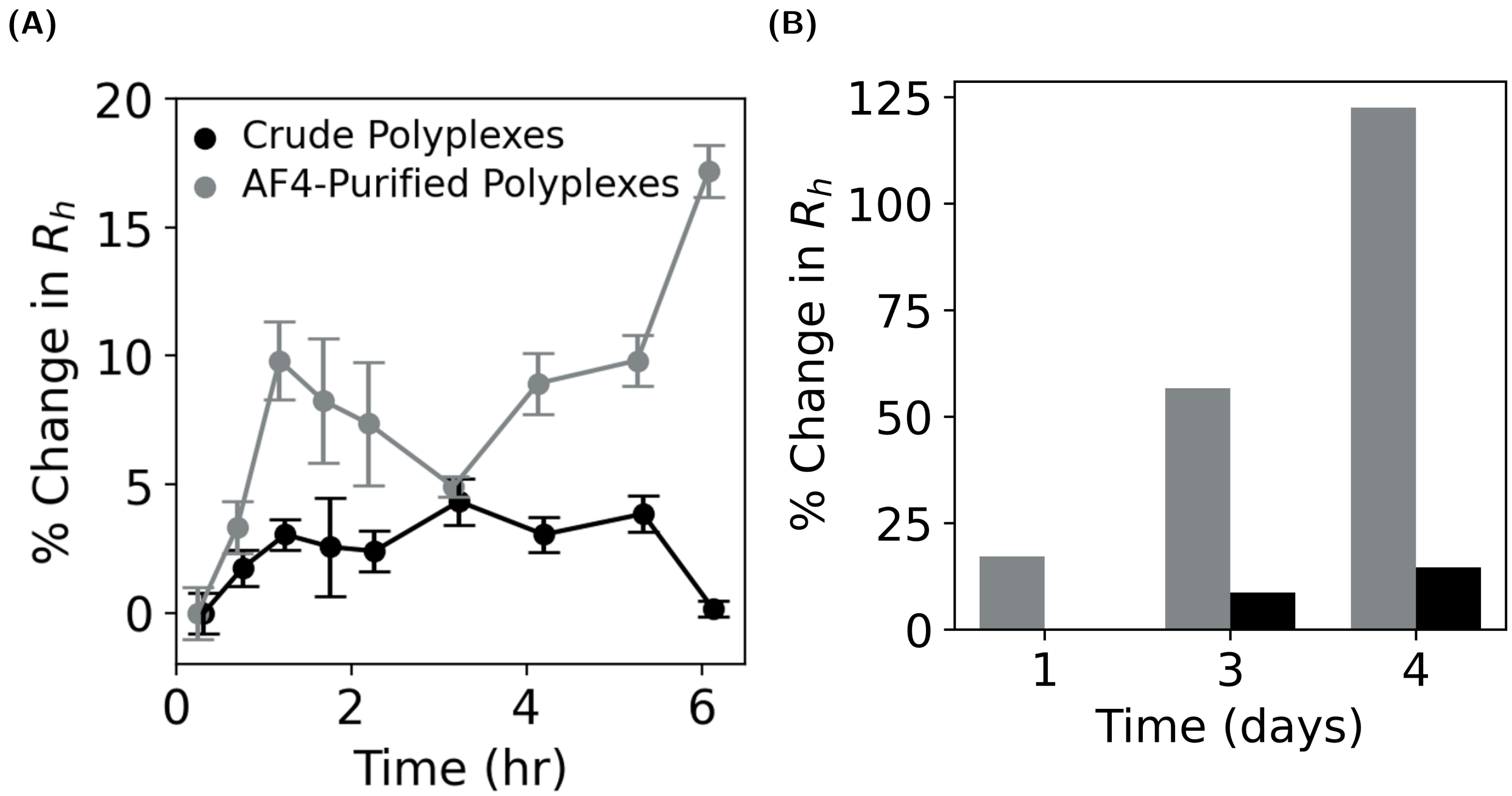

Figure S13. We assessed the stability of AF4-purified polyplexes (gray) by measuring the hydrodynamic radii (R_h) across multiple time points compared to crude polyplexes (black). (A) Both crude and AF4-purified polyplexes remained stable over 6 h. (B) AF4-purified polyplexes began to aggregate over later time points (days 3 and 4). Free polymers in the crude samples introduce electrostatic repulsion between polyplexes, preventing aggregation. Removing free polymers compromises long-term colloidal stability.

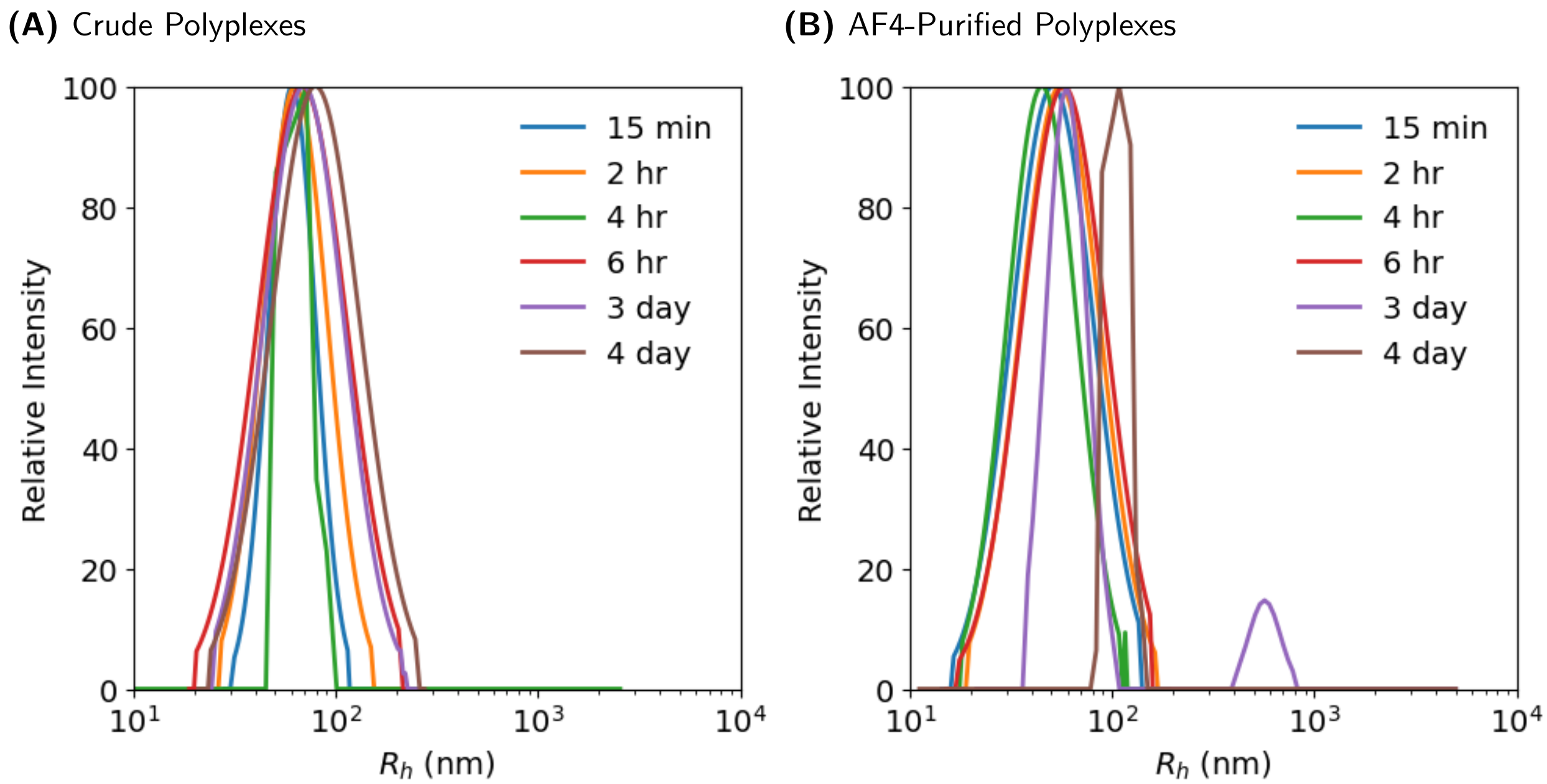

Figure S14. DLS histograms of crude and AF4-purified polyplexes collected at different time intervals from our stability study. (A) The hydrodynamic radius R_h of crude **S** polyplexes remained unchanged over the course of 4 days. (B) The AF4-purified polyplexes were stable over 6 h. On days 3 and 4, multimodal distributions of the polyplex radii began to appear, indicating polyplex aggregation. By day 4, the average polyplex hydrodynamic radius was double its initial value.

11 Supplemental pDNA delivery results

(A) Crude Polyplexes

(B) AF4-Purified Polyplexes

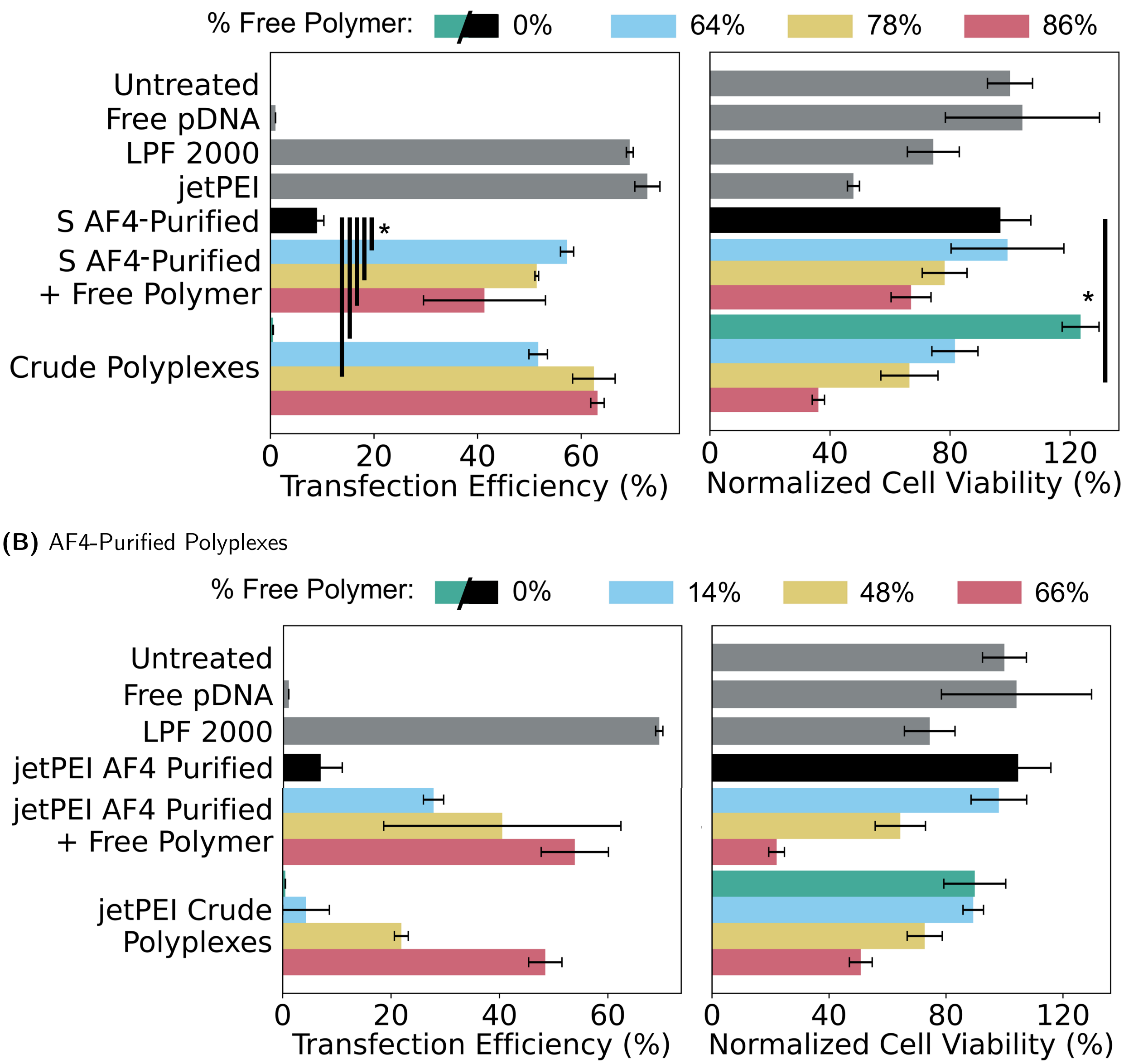

Figure S15. We compared the transfection efficiency and cytotoxicity of crude and AF4-purified polyplexes. (A) Data from control groups added to Figure 5 of the main manuscript. (B) AF4-purified jetPEI polyplexes were obtained by separating free jetPEI from crude polyplexes formed at N/P ratio of 5. AF4-purified polyplexes showed greatly reduced transfection efficiency and were better tolerated by cells compared to crude polyplexes at an N/P ratio of 5. Upon reintroducing free jetPEI polymers into the AF4-purified polyplexes, we restored the transfection efficiency and toxicity of the crude polyplexes.

12 Immunoassay flow cytometry gating schemes

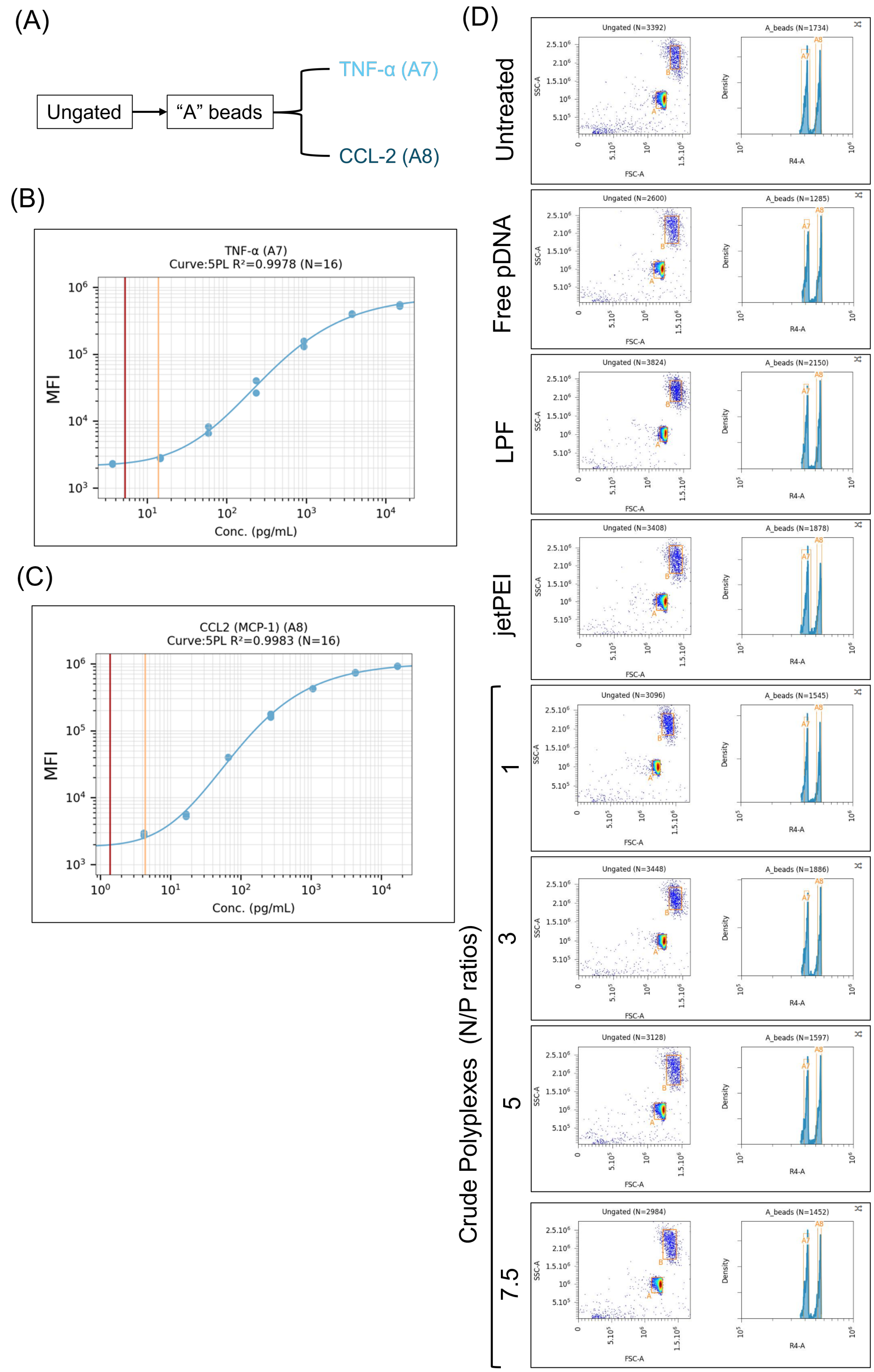

Figure S16. Flow cytometry gating schemes for immunoassay in Figure 4 of the main manuscript.

References

(1) Lawson, J. L.; Sekar, R. P.; Wright, A. R.; Wheeler, G.; Yanes, J.; Estridge, J.; Johansen, C. G.; Farnsworth, N. L.; Kumar, P.; Tay, J. W.; Kumar, R. The Spatial Distribution of Lipophilic Cations in Gradient Copolymers Regulates Polymer–pDNA Complexation, Polyplex Aggregation, and Intracellular pDNA Delivery. *Biomacromolecules* **2024**,

(2) Hu, Y. et al. Kinetic Control in Assembly of Plasmid DNA/Polycation Complex Nanoparticles. *ACS Nano* **2019**, *13*, 10161–10178.

(3) Shan, L.; Morris, V. B.; Labhasetwar, V. Co-Delivery of DNA and siRNA via Arginine-Rich PEI-based Polyplexes. *Mol. Pharmaceutics* **2015**, *12*, 621–629.

(4) D'Andrea, C.; Pezzoli, D.; Malloggi, C.; Candeo, A.; Capelli, G.; Bassi, A.; Volonterio, A.; Taroni, P.; Candiani, G. The Study of Polyplex Formation and Stability by Time-Resolved Fluorescence Spectroscopy of SYBR Green I-stained DNA. *Photochem. Photobiol. Sci.* **2014**, *13*, 1680–1689.
